# Determining *Streptococcus suis* serotype from short-read whole-genome sequencing data

**DOI:** 10.1186/s12866-016-0782-8

**Published:** 2016-07-22

**Authors:** Taryn B. T. Athey, Sarah Teatero, Sonia Lacouture, Daisuke Takamatsu, Marcelo Gottschalk, Nahuel Fittipaldi

**Affiliations:** Public Health Ontario Toronto Laboratory, 661 University Avenue, Toronto, ON M5G 1M1 Canada; Groupe de Recherche sur les Maladies Infectieuses du Porc, Faculty of Veterinary Medicine, University of Montreal, St-Hyacinthe, QC Canada; Bacterial and Parasitic Diseases Research Division, National Institute of Animal Health, National Agriculture and Food Research Organization, Tsukuba, Japan; The United Graduate School of Veterinary Sciences, Gifu University, Gifu, Japan; Department of Laboratory Medicine and Pathobiology, Faculty of Medicine, University of Toronto, Toronto, ON Canada

**Keywords:** *Streptococcus suis*, Serotyping, Whole-genome sequencing, Short-reads

## Abstract

**Background:**

*Streptococcus suis* is divided into 29 serotypes based on a serological reaction against the capsular polysaccharide (CPS). Multiplex PCR tests targeting the *cps* locus are also used to determine *S. suis* serotypes, but they cannot differentiate between serotypes 1 and 14, and between serotypes 2 and 1/2. Here, we developed a pipeline permitting *in silico* serotype determination from whole-genome sequencing (WGS) short-read data that can readily identify all 29 *S. suis* serotypes.

**Results:**

We sequenced the genomes of 121 strains representing all 29 known *S. suis* serotypes. We next combined available software into an automated pipeline permitting *in silico* serotyping of strains by differential alignment of short-read sequencing data to a custom *S. suis cps* loci database. Strains of serotype pairs 1 and 14, and 2 and 1/2 could be differentiated by a missense mutation in the *cpsK* gene*.* We report a 99 % match between coagglutination- and pipeline-determined serotypes for strains in our collection. We used 375 additional *S. suis* genomes downloaded from the NCBI’s Sequence Read Archive (SRA) to validate the pipeline. Validation with SRA WGS data resulted in a 92 % match. Included pipeline subroutines permitted us to assess strain virulence marker content and obtain multilocus sequence typing directly from WGS data.

**Conclusions:**

Our pipeline permits rapid and accurate determination of *S. suis* serotype, and other lineage information, directly from WGS data. By discriminating between serotypes 1 and 14, and between serotypes 2 and 1/2, our approach solves a three-decade longstanding *S. suis* typing issue.

**Electronic supplementary material:**

The online version of this article (doi:10.1186/s12866-016-0782-8) contains supplementary material, which is available to authorized users.

## Background

*Streptococcus suis* is a major swine pathogen responsible for severe economic losses to the porcine industry, and an emerging zoonotic agent of disease [1, 2]. *S. suis* strains are typed based on a serological reaction against the capsular polysaccharide (CPS), whose biosynthesis machinery is encoded by genes located in the *cps* locus [3, 4]. Thirty-five different *S. suis* serotypes were originally described (serotypes 1–34 and serotype 1/2) [5]. However, serotypes 20, 22, 26, 33, 32 and 34 have recently been shown or hypothesized to belong to novel bacterial species [6, 7]. Therefore, the *S. suis* species currently comprises a total of 29 serotypes. Accurate *S. suis* serotype identification is of significant epidemiological importance for the control of swine infections, since different serotypes are prevalent in different parts of the world [8]. Although it is widely considered the gold standard for typing, serology is expensive and time-consuming, and can only be performed at select reference laboratories. In addition, serology cannot readily discriminate between serotypes 2 and 1/2, or between serotypes 1 and 14, which cross-react on the coagglutination test [9]. Recently, elaborated PCR schemes targeting the *cps* locus permitting rapid “serotyping” of *S. suis* strains were described [10, 11]. However, due to the similar gene content of their respective *cps* loci, these PCR schemes do not differentiate between serotypes 2 and 1/2, and between serotypes 1 and 14 [10, 11]. Accurately distinguishing between serotypes 2 and 1/2, as well as between 1 and 14, is important since, based on current data, serotypes 2 and 14 are heavily associated with zoonotic disease [12].

Recent advances in whole-genome sequencing (WGS) technologies now permit the sequencing of hundreds of bacterial genomes rapidly and relatively inexpensively. WGS approaches are revolutionizing microbial typing in both the human and veterinary fields, and enhancing public health objectives such as disease surveillance, epidemic investigation and infection control [13–15]. In addition to typing, WGS data analysis permits rapid identification of known and putative virulence factors present in the genomes of the strains of interest [16, 17]. Here we report the development of a user-friendly bioinformatics pipeline running on the Linux operating system that combines available free software, and which can be used for rapid determination of *S. suis* serotype directly from the short-read WGS data. The pipeline correctly discriminates between all 29 *S. suis* serotypes, and permits to differentiate between serotypes 1 and 14, and between serotypes 2 and 1/2. The pipeline also uses the short-read WGS data to determine strain multilocus sequence typing (MLST) sequence type (ST) and virulence factor content.

## Methods

### Whole-genome sequencing

We sequenced the genomes of 121 *S. suis* strains belonging to 29 different serotypes (Additional file 1: Table S1). The strains used in this study have not been collected specifically for this study. They were part of the author’s extensive collection of clinical isolates. Serotyping was performed using the coagglutination test as previously described [9]. Strains were grown overnight in Todd-Hewitt broth (BD Bioscience, San Jose, CA) supplemented with 0.2 % yeast extract at 37 °C with 5 % CO_2_. DNA was prepared using the QIAamp DNA minikit (Qiagen, Toronto, Canada) following the manufacturer’s protocol for Gram-positive organisms. Genomic libraries were prepared using Nextera XT kits (Illumina, San Diego, CA) and sequenced as paired-ends using either a HiSeq 2500 (125 bp + 125 bp) or a MiSeq (150 bp + 150 bp) instruments (Illumina). Generated data was deposited in the Sequence Read Archive (SRA) under the Accession Numbers provided in Additional file 1: Table S1.

### Capsular typing pipeline

Our pipeline automates the execution of software that aligns WGS short-reads to custom or available *S. suis* sequence databases and permits subsequent sequence comparisons. Briefly, the nucleotide sequence of gene *recN*, which is unique to the *S. suis* species [18], was retrieved from the genome of *S. suis* serotype 2 strain NSUI002 (GenBank Accession Number CP011419), and included as *S. suis* species control. Short-read WGS data were then aligned to the *recN* sequence using the program SRST2, a software originally conceived as a means to derive MLST information from bacterial short-read WGS data [19]. However, SRST2 can be used to derive any typing information of interest if an appropriate sequence database is provided, as exemplified by reported pipelines permitting *emm* typing of Group A *Streptococcus* from short-read WGS data [13]. We next created a *S. suis cps* database by first downloading from GenBank sequences corresponding to each of the 27 *S. suis cps* loci differentiable by a multiplex PCR (Additional file 1: Table S2). Serotype specific *cps* loci regions were defined using *in silico* PCR and the primer sequences described in a multiplex PCR assay designed to differentiate *S. suis* serotypes [20]. Specific sequences for serotype defining amplicons were extracted using custom scripts. After species confirmation, our pipeline uses SRST2 to align short-read WGS data to the abovementioned *cps* database and makes an initial serotype assignment. Serotype pairs 1 and 14, and 2 and 1/2 cannot be identified directly using SRST2 (a limitation of the multiplex PCR assay, and thus of our custom *cps* amplicon database). Therefore, to resolve these ambiguous results, our pipeline next uses custom scripts that identify single-nucleotide polymorphisms in the *cps2K* gene of every strain identified as a putative serotype 1 or 14, or as putative serotype 2 or 1/2, followed by translation of the amino acid sequences for each strain. Based on the amino acid present at position 161 of the predicted translated sequence of the *cpsK* gene, the pipeline assigns the final serotype for the strain.

### Sanger sequencing of the *cpsK* locus

In order to confirm findings for the differences in the *cpsK* gene, we amplified a fragment of interest from DNA of 13 additional serotype 1/2 strains using primers cpsK-F (5’-GTTGCTGGTTATGATAGGGTAG-3’) and cpsK-R (5’-AACTCATAGAGCAAGCGATAAG-3’), followed by Sanger sequencing of the generated amplicon using the same primers.

### MLST, virulence marker gene content, and validation of the pipeline

The *S. suis* MLST database was downloaded from the *S. suis* MLST website available at ssuis.mlst.net. Sequence types (STs) were determined directly from the short-read WGS data using SRST2 [19]. The sequences of virulence marker genes *epf*, *sly*, and *mrp* were compared between multiple strains, and conserved subsequences were extracted. The presence or absence of these virulence factors was determined based on short-read alignment to these conserved sequences using SRST2. Pipeline partial outputs (*recN*, *cps* locus, MLST and virulence marker content) were then combined in a final output file. To validate our pipeline, previously generated WGS short-read data for 367 *S. suis* strains were downloaded from the SRA (Additional file 1: Table S3).

## Results and Discussion

### Species confirmation

**Fig. 1:**
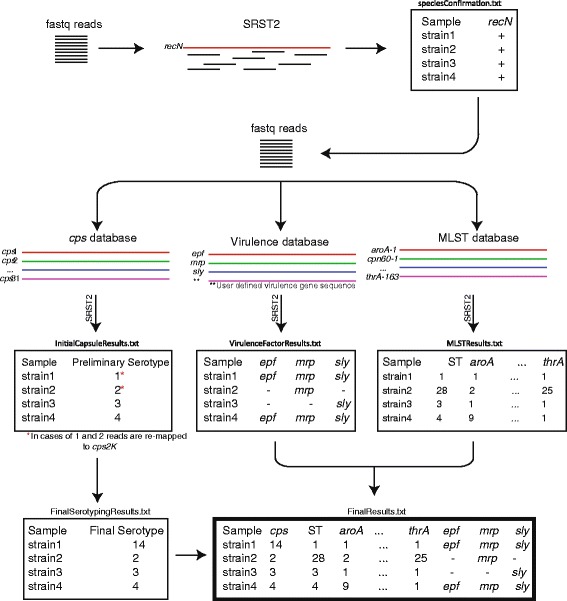
Schematic of the *S. suis* serotyping pipeline. The pipeline takes in *S. suis* WGS reads in fastq format and first confirms that the isolate belongs to the species *S. suis* by aligning reads to the gene *recN* using SRST2 software. Once species has been confirmed, SRST2 is invoked in three parallel instances to identify serotype, MLST information, and virulence gene content using provided databases. The serotyping component cannot differentiate between serotype 1 and serotype 14, or between serotype 2 and serotype 1/2 without additional comparison of the sequences of the *cps2K* gene (see results). Final serotype determination results are then combined with MLST and virulence results to provide the user a single output file

We sequenced the genomes of 121 *S. suis* strains representing 29 different serotypes. The coverage across the genome was on average 56X, considering an average *S. suis* genome size of 2.2 Mbp (Additional file 1: Table S1). An overview of our pipeline is presented in Fig. 1. Essentially, the pipeline uses SRST2 [19] to align the short-read WGS data to various databases that are modifiable. In a first iteration, short-reads are aligned against the sequence of the gene *recN*. All 121 strains were positive for this gene. Although the specificity of this gene for the *S. suis* species has been verified extensively [18], here we assessed *recN* specificity using short-read WGS generated in our laboratories for 1,040 strains of *Streptococcus agalactiae*, *Streptococcus pyogenes*, *Streptococcus pneumoniae*, *Streptococcus pluranimalium*, *Streptococcus porcinus*, *Actinobacillus pleuropneumoniae* and *Listeria monocytogenes*. None of the genomes of the strains of these species were *recN* positive (data not shown).

### Capsular typing from short-read WGS

Next, our pipeline aligns short-read WGS data to a custom *cps* database. The *cps* database is constructed based on expected amplicons defined by Kerdsin *et al.* [20]. Since this database cannot differentiate between serotypes 1 and 14 or between serotypes 2 and 1/2, it represents 27 of the currently accepted 29 *S. suis* serotypes. At this step, our pipeline is also unable to discriminate between these two serotype pairs. A total of 120 strains were assigned a serotype, or putative serotype in the case of the abovementioned serotype pairs by short-read alignment to the custom *cps* database (Additional file 1: Table 1). One strain, typed by coagglutination as serotype 8, was not assigned a serotype by our pipeline, i.e., it was designated as “non-typable”. To investigate this unexpected result, we manually inspected the BAM file containing the alignment of the reads of this strain to the *cps8* locus. Manual inspection revealed several areas of very low short-read coverage. Alignment of reads of this strain to *de novo* genome assemblies for other serotype 8 strains generated using the A5 pipeline [21] revealed that coverage across the genome of this non-typable strain was uneven (biased, likely the result of suboptimal tagmentation during library preparations), and some areas of the *cps8* locus had a coverage depth of 1X (data not shown). The program SRST2 requires, by default, a read coverage of at least 3X in order to identify an allele [19]. Thus, this result exemplifies one limitation of our pipeline, which is that adequate and even short-read coverage is necessary to confidently identify serotypes. Resequencing of the genome of this isolate permitted us to reach a coverage of 331 X. With these novel data, the pipeline correctly assigned the isolate to serotype 8. As a general rule, we recommend that users of our pipeline generate WGS data with an across-genome read coverage of at least 30X, which is in accordance with the American College of Medical Genetics and Genomics recommendations for the use of next-generation sequencing in clinical laboratories [22]. Alternatively, in cases where a serotype is not assigned, expert users that have low coverage samples for which a positive *recN* result is obtained could manually inspect the BAM files reporting alignment to each type-specific *cps* sequence before concluding the strain is non-typable.

**Table 1:** Serotype determination using the *S. suis* serotyping pipeline

Serotype	Number of strains with this serotype on the coagglutination test	Number of strains identified in pipeline first pass	Number of strains identified in pipeline second pass	Accuracy After Second Pass
1/2	7	N/A^a^	7	100 %
1	7	14	7	100 %
2	7	14	7	100 %
3	5	5	5	100 %
4	7	7	7	100 %
5	7	7	7	100 %
6	1	1	1	100 %
7	7	7	7	100 %
8	6	5^b^	5^b^	83 %^b^
9	9	9	9	100 %
10	2	2	2	100 %
11	1	1	1	100 %
12	1	1	1	100 %
13	1	1	1	100 %
14	7	N/A	7	100 %
15	1	1	1	100 %
16	7	7	7	100 %
17	2	2	2	100 %
18	2	2	2	100 %
19	3	3	3	100 %
21	2	2	2	100 %
23	4	4	4	100 %
24	4	4	4	100 %
25	1	1	1	100 %
27	2	2	2	100 %
28	6	6	6	100 %
29	2	2	2	100 %
30	7	7	7	100 %
31	3	3	3	100 %

^a^N/A: not applicable; at this stage serotype 1/2 strains are presumptively assigned to serotype 2 and serotype 14 strains are presumptively assigned to serotype 1

^b^All six serotype 8 strains were assigned to serotype 8 by our pipeline after resequencing of the genome of one serotype isolate whose genome had originally been sequenced at low read coverage due to technical issues during genomic libraries preparation

### Differentiating between serotypes 1 and 14 and between serotypes 2 and 1/2

**Fig. 2:**
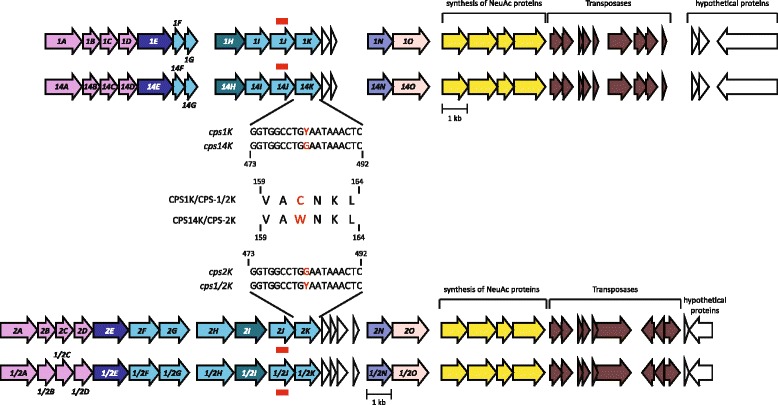
Schematics of *cps* loci and identification of a single-nucleotide polymorphism permitting differentiation of the *S. suis* serotypes 1 and 14, and 2 and 1/2. The *cps* loci of serotypes 1 and 14 are identical in gene content (top), as are the *cps* loci of serotypes 2 and 1/2 (bottom). However, analysis of all available serotype 1, 14, 2 and 1/2 strains in our collection revealed that serotype 14 strains consistently differed from serotype 1 strains, and that serotype 2 strains consistently differed from serotype 1/2 strains by a single-nucleotide change (G to T or C) in the *cpsK* gene, which is predicted to result in an amino acid change from tryptophan to cysteine. Our pipeline uses this amino acid change to differentiate between those otherwise unresolvable serotype pairs and assign the serotype to the strain under investigation. Red bars indicate regions used in the first step of the pipeline to differentiate serotypes 1 and 14 as well as serotypes 2 and 1/2 from other serotypes

Genetic studies have shown that the *cps* loci of *S. suis* serotypes 1 and 14 have exact gene content [23]. Similarly, the *cps* loci of serotypes 2 and 1/2 strains have the same gene content [23]. We hypothesized that differences exist at the single-nucleotide level in the *cps* loci of those pairs of serotypes, and that those differences permit to resolve each serotype pair into individual types. To test the initial hypothesis, we *de novo* assembled the short-reads of all 7 isolates of each of these serotypes included in our collection using the A5 pipeline [21]. The *de novo* assemblies were screened to identify each *cps* loci, which were then aligned using ClustalW [24] and interrogated for polymorphisms unique to each serotype. The analysis revealed that all serotype 2 and all serotype 14 strains had a G nucleotide at position 483 of the *cpsK* gene, while all serotype 1 and all serotype 1/2 strains contained either a C or T at that nucleotide position. Thus, the predicted sequence of the CpsK protein in serotypes 2 and 14 possess amino acid tryptophan at position 161, while the predicted sequence of the CpsK protein in serotypes 1/2 and 1 has amino acid cysteine at this position (Fig. 2). The genome sequence of a serotype 1/2 strain from China has been published [25]. When we examined the reported sequence of the *cpsK* gene of this Chinese strain (GenBank Accession Number NC_017619), we did not find the abovementioned polymorphism, i.e., the *cpsK* gene of the Chinese strain has a G nucleotide at position 483 resulting in a TGG codon. Since this reported *cpsK* sequence raises concerns about the universality of the polymorphism permitting differentiation of serotypes 2 and 1/2 identified in this study, and in order to study the issue in more detail, we performed Sanger sequencing of the *cpsK* gene of 13 additional serotype 1/2 strains recovered in Canada. In agreement with our genomic findings, Sanger sequencing identified that the c*psK* gene of these additional serotype 1/2 strains contained a T nucleotide at position 483. Moreover, when we used external WGS data to validate our pipeline (see below), none of the 10 genomes of serotype 1/2 strains reported in a previous publication [26] had a *cpsK* gene sequence similar to the serotype 1/2 Chinese strain, but, instead, they had one of the polymorphisms described here. CpsK is a glycosyltransferase predicted to add galactose (in serotypes 2 and 14) or N-acetylgalactosamine (in serotypes 1 and 1/2) to the polysaccharide chain [27–29]. We speculate that the polymorphism at amino acid position 161 of CpsK is critical in defining the sugar that this enzyme recognizes. Using targeted mutagenesis (replacement of CpsK amino acid residue at position 161 from W to C), we have very recently been able to switch the capsular polysaccharide from 2 to 1/2 in a serotype 2 strain. Conversely, after inverting the substitution, i.e. C to W substitution, we transformed a serotype 1/2 strain into a serotype 2 strain (Roy *et al.*, manuscript in preparation). We thus believe that either the reported genome sequence of the Chinese serotype 1/2 strain contains an error in the *cpsK* gene sequence or that the strain was misidentified as serotype 1/2 when it is, in fact, serotype 2. Unfortunately, short-reads for the Chinese strain are not available to confirm this speculation.

Our pipeline takes advantage of the abovementioned polymorphisms in *cpsK* to discriminate between these otherwise unresolvable pairs of serotypes. Briefly, after alignment to the custom *cps* database, the pipeline assigns a presumptive label of “serotype 1” to the potential pair 1–14 and a presumptive label of “serotype 2” to the potential pair 2-1/2. Next, it invokes a second mapping step, which by aligning short-reads of the strain under investigation to *cps2K* sequences, identifies SNPs and outputs the predicted amino acid sequence of CpsK. This amino acid sequence is then scanned for the presence of a tryptophan or a cysteine amino acid residue at position 161. A final serotype assignment (1 or 14, or 2 or 1/2) is then made based on the predicted translated codon. Using this second step, our pipeline matched coagglutination results in 120 cases (all strains, with the exception of the abovementioned serotype 8 strain; Table 1).

### Validation of the pipeline with external datasets

Next, we sought to validate our pipeline using available WGS datasets generated by others [26]. To this end, we downloaded from the SRA WGS short-read data for 375 *S. suis* strains. Of them, 329 represented 13 serotypes (Additional file 1: Table S3), whereas 46 strains had been described as non-typable by serology [26]. Based on the presence of serotype specific sequences, we were able to assign a serotype to 38 of the 46 non-typable strains using our pipeline. It must be noted, however, that serotype assignment by our pipeline cannot predict whether a strain actually expresses capsular material. As reported previously [30], mutations in *cps* loci genes, which occur frequently and which our pipeline is not designed to detect, as well as insertion of mobile genetic elements in the *cps* locus of any given *S. suis* strain, may lead to the absence of capsule expression. However, serotype determination of unencapsulated strains is useful to understand a variety of strain traits, including their origin and evolutionary path. The remaining eight strains present in the external collection that were reported as non-typable [26] did not match any of the sequences in our *cps* database, and thus were also identified as non-typable by our pipeline. The genomes of these eight strains were *de novo* assembled and BLASTN comparisons against the reference sequences of all 29 *cps* loci were carried out. We were unable to identify homology to any of the 29 *cps* loci. Thus, we conclude that these eight strains may represent novel *S. suis* serotypes.

Our pipeline and previously reported serotyping results [26] agreed in 303 of the 329 strains in the external collection (92 %). We examined the 26 mismatched isolates by manual inspection of BAM files containing the short-read data alignments to each of the specific *cps* regions defining serotypes in our database. The analysis revealed very poor short-read alignment to sequences for the loci encoding the previously reported serologically-determined serotypes [26]. On the other hand, in 22 out of these 26 cases, BAM inspection revealed very good coverage relative to the serotype-specific sequences corresponding to the serotype identified by our pipeline. However, in 4 cases, BAM file examination did not show good alignments to any of the sequences in the database. To unambiguously determine the quality of our pipeline results, WGS short-read data for these 26 strains were *de novo* assembled and local BLASTN comparisons were performed to identify the *cps* loci of the strains. In 22 cases, the strains had the *cps* loci expected for the serotype identified by our pipeline (Additional file 1: Figures S1 to S22), and not the one expected based on the reported serological results [26]. The remaining four strains were identified as non-typable by our pipeline. We were unable to assign any of these four strains to any known *S. suis* serotype based on BLASTN comparisons to known *cps* loci of the *de novo* assemblies (Additional file 1: Figures S23 to S26). Therefore, we conclude that these 4 strains are *bona fide* nontypable *S. suis* strains, while the 26 other strains had been mistyped by the serological method(s) used to ascertain their serotype [26].

### MLST determination and virulence factor content

Using the MLST database for *S. suis*, our pipeline next invokes SRST2 to determine the MLST STs of each strain (Additional file 1: Table S1). The vast majority of the strains were assigned to known STs already described in the *S. suis* MLST database, but we also identified 41 novel STs. Our pipeline was also used to determine presence or absence of virulence factors (Additional file 1: Table S1). The virulence factor database provided with our pipeline was designed as a proof of principle, and only screens for conserved regions of *mrp*, *epf* and *sly* genes, encoding a muramidase-released protein, an extracellular protein factor, and a hemolysin known as suilysin, respectively [31–34]. Users may add any virulence gene of interest to the pipeline virulence database. Results are presented in Additional file 1: Table S1. Our virulence results for *mrp*, *epf* and *sly* matched those described in previous studies in serotypes 1, 1/2, 2, 3, 7, and 9 [35, 36], which so far have been studied in a more consistent fashion than other *S. suis* serotypes.

## Conclusions

Serotyping of *S. suis* strains is crucial to infection control efforts and to reduce the possibility of zoonotic disease outbreaks [1, 2]. The need to accurately differentiate between serotypes 1 and 14, and between serotypes 2 and 1/2 is emphasized by the zoonotic potential of serotypes 2 and 14 [37]. Here, we show that WGS short-read data, which now can be generated rapidly and relatively inexpensively for large numbers of strains, can be used to precisely identify the serotype of *S. suis* strains, as well as to obtain other relevant strain information in an automated fashion. One limitation of our pipeline, which is common to previously reported multiplex PCR tests targeting the *cps* locus [10, 11], is that we only analyzed one or a few representative strains of rare serotypes such as 6, 11, 15, 21, 25*.* Analysis of a more important number of strains of these rare serotypes, when they become available, should increase the confidence in a pipeline positive result for these serotypes. Importantly, our pipeline can differentiate with high accuracy between serotypes 1 and 14, as well as between serotypes 2 and 1/2, which cannot be resolved by available multiplex PCR assays, or which require multiple serological reactions using specific antisera available only at a few selected reference laboratories worldwide. Compared to serology, one limitation of our WGS approach is that it does not identify whether a strain actually expresses a capsule. However, insights gained by determining the *cps* locus type of serologically non-typable strains are important for phylogenetic and evolution purposes. As demonstrated here when validating our pipeline with external data, it is not uncommon that *S. suis* isolates are mistyped using serological assays. Thus, using our pipeline reduces the variability introduced by different personnel performing such serological tests. If strains must be confirmed by using serological means, our pipeline can pinpoint the specific serotype that needs to be tested, thus saving time and money by eliminating the need for multiple serological reactions. The pipeline is open source and available for download from https://github.com/streplab/SsuisSerotyping_pipeline.

## Abbreviations

CPS, capsular polysaccharide; MLST, multi-locus sequence type; PCR, polymerase chain reaction; SNP, single nucleotide polymorphism; SRA, sequence read archive; SRST2, short read sequencing typing version 2; ST, sequence type; WGS, whole genome sequencing

## Additional file

Additional file 1:
*S. suis* strains used in this study; serotypes and accession numbers for reference capsule type sequences used in this study; information and pipeline results of S. suis strains downloaded from the Sequence Read Archive and comparison of cps loci of selected S. suis strains This supplementary file contains: 1) three supplementary Tables, 2) 26 supplementary Figures. (PDF 7086 kb)
