# Supplementary material for: Determining Streptococcus suis serotype from short-read whole-genome sequencing data
Source: BMC Microbiol. 2016 Jul 22;16:162. doi: 10.1186/s12866-016-0782-8 (PMC4957933; doi:10.1186/s12866-016-0782-8)
Supplement: Additional file 1: — S. suis strains used in this study; serotypes and accession numbers for reference capsule type sequences used in this study; information and pipeline results of S. suis strains downloaded from the Sequence Read Archive and comparison of cps loci of selected S. suis strains This supplementary file contains: 1) three supplementary Tables, 2) 26 supplementary Figures. (PDF 7086 kb) [file 12866_2016_782_MOESM1_ESM.pdf]

Supplementary Materials:

This supplementary file contains: 1) three supplementary Tables, 2) 26 supplementary Figures.

Table S1. ***Streptococcus suis*** **strains used in this study.**

Strain	Origin	Serotype		Sequence Type[b]	*aroA*	*cpn60*	*dpr*	*gki*	*mutS*	*recA*	*thrA*	*mrp*	*epf*	*sly*	*recN*	Illumina Sequence Data		SRA Accession Nº
		Coag[a]	Pipeline													Nº of Reads	Coverage	
NSUI117	Canada	1	1	1	1	1	1	1	1	1	1	+	+	+	+	916,102	52.05	SRR3066639
NSUI118	Canada	1	1	1	1	1	1	1	1	1	1	+	+	+	+	820,660	46.63	SRR3066640
NSUI119	Canada	1	1	1	1	1	1	1	1	1	1	+	+	+	+	851,154	48.36	SRR3066736
NSUI120	Canada	1	1	13	1	12	1	1	6	21	21	-	-	+	+	803,916	45.68	SRR3066747
NSUI121	Canada	1	1	1	1	1	1	1	1	1	1	+	+	+	+	982,266	55.81	SRR3066758
NSUI122	Canada	1	1	1	1	1	1	1	1	1	1	+	+	+	+	795,544	45.20	SRR3066769
NSUI123	Canada	1	1	1	1	1	1	1	1	1	1	+	+	+	+	1,009,884	57.38	SRR3066780
NSUI124	Canada	1/2	1/2	28	2	30	5	34	31	3	25	+	-	-	+	901,988	51.25	SRR3066791
NSUI125	Canada	1/2	1/2	28	2	30	5	34	31	3	25	+	-	-	+	1,164,954	66.19	SRR3066802
NSUI126	Canada	1/2	1/2	28	2	30	5	34	31	3	25	+	-	-	+	945,366	53.71	SRR3066813
NSUI127	Canada	1/2	1/2	28	2	30	5	34	31	3	25	+	-	-	+	1,038,676	59.02	SRR3066641
NSUI128	Canada	1/2	1/2	28	2	30	5	34	31	3	25	+	-	-	+	987,342	56.10	SRR3066684
NSUI129	Canada	1/2	1/2	28	2	30	5	34	31	3	25	+	-	-	+	655,476	37.24	SRR3066727
NSUI130	Canada	1/2	1/2	28	2	30	5	34	31	3	25	+	-	-	+	755,072	42.90	SRR3066729
NSUI131	Canada	2	2	25	9	30	5	34	30	3	25	+	-	-	+	915,430	52.01	SRR3066730
NSUI132	Canada	2	2	25	9	30	5	34	30	3	25	+	-	-	+	765,442	43.49	SRR3066731
NSUI133	Canada	2	2	28	2	30	5	34	31	3	25	+	-	-	+	1,171,864	66.58	SRR3066732
NSUI134	Canada	2	2	28	2	30	5	34	31	3	25	+	-	-	+	1,149,098	65.29	SRR3066733
NSUI135	Canada	2	2	25	9	30	5	34	30	3	25	+	-	-	+	1,315,186	74.73	SRR3066734
NSUI136	Canada	2	2	28	2	30	5	34	31	3	25	+	-	-	+	936,940	53.24	SRR3066735
NSUI137	Canada	2	2	25	9	30	5	34	30	3	25	+	-	-	+	919,264	52.23	SRR3066737
NSUI138	Canada	3	3	108	8	21	5	45	44	22	23	+	-	+	+	1,010,030	57.39	SRR3066738
NSUI139	Canada	3	3	Novel[c]	8	21	5	34	44	22	23	+	-	+	+	795,330	45.19	SRR3066739
NSUI140	Canada	3	3	117	31	57	5	34	31	3	25	+	-	-	+	990,586	56.28	SRR3066740
NSUI141	Canada	3	3	27	31	30	5	34	31	3	25	+	-	-	+	967,290	54.96	SRR3066741
NSUI142	Canada	3	3	27	31	30	5	34	31	3	25	+	-	-	+	1,082,054	61.48	SRR3066742
NSUI143	Canada	4	4	94	8	21	5	45	44	22	4	+	-	+	+	1,118,392	63.55	SRR3066743
NSUI144	Canada	4	4	Novel	8	21	5	45	44	22	109	+	-	+	+	897,722	51.01	SRR3066744
NSUI145	Canada	4	4	94	8	21	5	45	44	22	4	+	-	+	+	803,550	45.66	SRR3066745
NSUI146	Canada	4	4	Novel	91	21	5	45	44	22	23	+	-	+	+	805,218	45.75	SRR3066746
NSUI147	Canada	4	4	94	8	21	5	45	44	22	4	+	-	+	+	1,032,564	58.67	SRR3066748

Strain	Origin	Serotype		Sequence Type[b]	*aroA*	*cpn60*	*dpr*	*gki*	*mutS*	*recA*	*thrA*	*mrp*	*epf*	*sly*	*recN*	Illumina Sequence Data		SRA Accession Nº
		Coag[a]	Pipeline													Nº of Reads	Coverage	
NSUI148	Canada	4	4	108	8	21	5	45	44	22	23	+	-	+	+	850,902	48.35	SRR3066749
NSUI149	Canada	4	4	94	8	21	5	45	44	22	4	+	-	+	+	1,023,312	58.14	SRR3066750
NSUI150	Canada	5	5	94	8	21	5	45	44	22	4	+	-	+	+	1,036,942	58.92	SRR3066751
NSUI151	Canada	5	5	89	8	8	24	12	1	10	4	+	-	+	+	769,192	43.70	SRR3066752
NSUI152	Canada	5	5	53	8	6	18	20	28	7	5	+	-	+	+	897,458	50.99	SRR3066753
NSUI153	Canada	5	5	235	57	76	53	6	98	15	76	-	-	-	+	846,396	48.09	SRR3066754
NSUI154	Canada	5	5	Novel	38	28	85	4	84	26	11	-	-	-	+	1,006,304	57.18	SRR3066755
NSUI155	Canada	5	5	Novel	38	28	85	4	84	26	49	-	-	-	+	1,111,124	63.13	SRR3066756
NSUI156	Canada	5	5	Novel	8	21	5	45	44	22	109	+	-	+	+	1,190,748	67.66	SRR3066757
NSUI157	Canada	6	6	55	25	37	15	41	15	23	10	-	-	-	+	1,064,592	60.49	SRR3066759
NSUI158	Canada	7	7	89	8	8	24	12	1	10	4	+	-	+	+	954,670	54.24	SRR3066760
NSUI159	Canada	7	7	225	8	30	5	34	44	21	4	-	-	+	+	1,035,986	58.86	SRR3066761
NSUI160	Canada	7	7	89	8	8	24	12	1	10	4	+	-	+	+	727,734	41.35	SRR3066762
NSUI161	Canada	7	7	94	8	21	5	45	44	22	4	+	-	+	+	789,048	44.83	SRR3066763
NSUI162	Canada	7	7	94	8	21	5	45	44	22	4	+	-	+	+	1,034,780	58.79	SRR3066764
NSUI163	Canada	7	7	Novel	8	21	5	45	44	22	109	+	-	+	+	872,236	49.56	SRR3066765
NSUI164	Canada	7	7	32	8	30	5	34	30	21	4	-	-	+	+	775,384	44.06	SRR3066766
NSUI165	Canada	8	NT[d] (8)	LC[e] (87)	18	8	24	LC (12)	1	10	4	+	-	+	+	337,516	19.18	SRR3066767[f]
NSUI166	Canada	8	8	87	18	8	24	12	1	10	4	+	-	+	+	561,586	31.91	SRR3066768
NSUI167	Canada	8	8	LC (87)	18	8	LC (24)	12	1	10	4	+	-	+	+	350,536	19.92	SRR3066770
NSUI168	Canada	8	8	LC (87)	LC (18)	LC (8)	24	12	1	LC (10)	4	+	-	+	+	215,674	12.25	SRR3066771
NSUI170	Canada	8	8	87	18	8	24	12	1	10	4	+	-	+	+	736,500	41.85	SRR3066772
NSUI171	Canada	8	8	87	18	8	24	12	1	10	4	+	-	+	+	600,254	34.11	SRR3066773
NSUI172	Canada	9	9	Novel	48	33	30	66	48	55	76	+	-	-	+	531,790	30.22	SRR3066774
NSUI173	Canada	9	9	Novel	17	21	5	45	44	22	25	+	-	+	+	872,068	49.55	SRR3066775
NSUI174	Canada	10	10	Novel	77	142	49	15	221	64	38	+	-	-	+	854,114	48.53	SRR3066776
NSUI175	Canada	9	9	16	5	17	5	12	1	10	4	+	-	+	+	935,604	53.16	SRR3066777
NSUI176	Canada	9	9	16	5	17	5	12	1	10	4	+	-	+	+	943,246	53.59	SRR3066778
NSUI177	Canada	9	9	16	5	17	5	12	1	10	4	+	-	+	+	843,796	47.94	SRR3066779
NSUI178	Canada	9	9	16	5	17	5	12	1	10	4	+	-	+	+	1,033,140	58.70	SRR3066781

Strain	Origin	Serotype		Sequence Type[b]	*aroA*	*cpn60*	*dpr*	*gki*	*mutS*	*recA*	*thrA*	*mrp*	*epf*	*sly*	*recN*	Illumina Sequence Data		SRA Accession Nº
		Coag[a]	Pipeline													Nº of Reads	Coverage	
NSUI179	Canada	25	25	Novel	23	68	21	39	14	6	8	-	-	-	+	1,137,366	64.62	SRR3066782
NSUI180	Canada	10	10	Novel	23	68	21	4	14	6	8	-	-	-	+	888,536	50.49	SRR3066783
NSUI181	Canada	12	12	Novel	147	124	130	176	216	106	Novel	-	-	-	+	1,043,610	59.30	SRR3066784
NSUI182	Canada	13	13	Novel	96	177	129	235	139	90	12	-	-	-	+	1,057,448	60.08	SRR3066785
NSUI183	Canada	14	14	1	1	1	1	1	1	1	1	+	+	+	+	1,018,224	57.85	SRR3066786
NSUI184	Canada	14	14	1	1	1	1	1	1	1	1	+	+	+	+	908,638	51.63	SRR3066787
NSUI185	Canada	14	14	28	2	30	5	34	31	3	25	+	-	-	+	947,042	53.81	SRR3066788
NSUI186	Canada	14	14	1	1	1	1	1	1	1	1	+	+	+	+	1,039,894	59.08	SRR3066789
NSUI187	Canada	14	14	1	1	1	1	1	1	1	1	+	+	+	+	973,144	55.29	SRR3066790
NSUI188	Canada	14	14	1	1	1	1	1	1	1	1	+	+	+	+	890,230	50.58	SRR3066792
NSUI189	Canada	14	14	1	1	1	1	1	1	1	1	+	+	+	+	1,114,954	63.35	SRR3066793
NSUI191	Canada	16	16	Novel	23	25	21	4	7	30	8	-	-	-	+	1,044,802	59.36	SRR3066794
NSUI192	Canada	16	16	Novel	23	68	21	4	14	6	8	-	-	-	+	965,198	54.84	SRR3066795
NSUI193	Canada	16	16	Novel	17	68	21	39	14	6	8	-	-	-	+	981,358	55.76	SRR3066796
NSUI194	Canada	16	16	Novel	63	15	45	4	16	68	12	-	-	-	+	1,229,950	69.88	SRR3066797
NSUI195	Canada	16	16	Novel	15	8	24	12	1	10	4	+	-	+	+	948,660	53.90	SRR3066798
NSUI196	Canada	16	16	Novel	23	190	21	4	14	6	8	-	-	-	+	1,087,292	61.78	SRR3066799
NSUI197	Canada	16	16	73	33	37	2	19	13	5	12	-	-	-	+	1,146,336	65.13	SRR3066800
NSUI198	Canada	17	17	76	8	23	36	24	21	22	12	+	-	+	+	1,181,714	67.14	SRR3066801
NSUI199	Canada	17	17	76	8	23	36	24	21	22	12	+	-	+	+	1,140,434	64.80	SRR3066803
NSUI200	Canada	18	18	Novel	8	21	36	24	42	22	12	-	-	+	+	1,005,268	57.12	SRR3066804
NSUI201	Canada	18	18	76	8	23	36	24	21	22	12	+	-	+	+	842,416	47.86	SRR3066805
NSUI202	Canada	19	19	76	8	23	36	24	21	22	12	+	-	+	+	1,180,026	67.05	SRR3066806
NSUI203	Canada	19	19	76	8	23	36	24	21	22	12	+	-	+	+	1,025,704	58.28	SRR3066807
NSUI204	Canada	19	19	Novel	8	21	36	24	42	22	12	-	-	+	+	906,570	51.51	SRR3066808
NSUI208	Canada	21	21	Novel	25	228	58	24	LC (145)	37	16	-	-	-	+	348,308	19.79	SRR3066809
NSUI218	Canada	23	23	108	8	21	5	45	44	22	23	+	-	+	+	836,914	47.55	SRR3066810
NSUI221	Canada	23	23	483	8	21	3	45	44	22	23	+	-	+	+	1,028,634	58.45	SRR3066811
NSUI222	Canada	23	23	108	8	21	5	45	44	22	23	+	-	+	+	1,181,352	67.12	SRR3066812
NSUI223	Canada	23	23	108	8	21	5	45	44	22	23	+	-	+	+	1,049,984	59.66	SRR3066814
NSUI224	Canada	24	24	68	14	20	31	27	27	34	18	-	-	-	+	1,127,460	64.06	SRR3066815
NSUI225	Canada	24	24	221	77	33	49	15	33	26	6	-	-	-	+	1,042,090	59.21	SRR3066816

Strain	Origin	Serotype		Sequence Type[b]	*aroA*	*cpn60*	*dpr*	*gki*	*mutS*	*recA*	*thrA*	*mrp*	*epf*	*sly*	*recN*	Illumina Sequence Data		SRA Accession №
		Coag[a]	Pipeline													№ of Reads	Coverage	
NSUI226	Canada	24	24	234	77	33	1	15	33	26	6	-	-	-	+	781,990	44.43	SRR3066817
NSUI227	Canada	24	24	Novel	14	58	39	155	222	75	110	-	-	-	+	1,043,698	59.30	SRR3066818
NSUI229	Canada	27	27	Novel	161	13	143	95	117	38	124	-	-	-	+	1,262,898	71.76	SRR3066819
NSUI230	Canada	27	27	Novel	91	29	36	95	117	38	98	-	-	-	+	1,431,466	81.33	SRR3066820
NSUI231	Canada	28	28	Novel	114	159	17	163	94	38	51	-	-	-	+	1,248,906	70.96	SRR3066821
NSUI232	Canada	28	28	Novel	178	58	58	155	82	34	8	-	-	-	+	1,292,106	73.42	SRR3066822
NSUI233	Canada	28	28	Novel	120	81	143	87	181	12	123	-	-	-	+	1,257,534	71.45	SRR3066823
NSUI234	Canada	28	28	Novel	188	240	119	103	193	39	51	-	-	-	+	764,750	43.45	SRR3066642
NSUI236	Canada	28	28	Novel	178	58	58	155	82	34	8	-	-	-	+	1,458,638	82.88	SRR3066643
NSUI237	Canada	28	28	Novel	203	152	36	24	21	38	12	-	-	-	+	1,395,948	79.32	SRR3066644
NSUI238	Canada	21	21	Novel	25	152	36	24	21	38	13	-	-	-	+	1,615,900	91.81	SRR3066645
NSUI239	Canada	29	29	Novel	147	78	132	139	159	116	115	-	-	-	+	1,340,866	76.19	SRR3066646
NSUI241	Canada	29	29	Novel	31	31	5	34	52	18	18	+	-	+	+	1,149,326	65.30	SRR3066647
NSUI242	Canada	30	30	Novel	62	14	72	62	216	42	8	-	-	-	+	1,008,998	57.33	SRR3066648
NSUI243	Canada	30	30	Novel	7	6	28	151	9	160	8	+	-	+	+	1,178,238	66.95	SRR3066649
NSUI244	Canada	30	30	Novel	91	4	176	103	82	36	92	-	-	-	+	1,405,816	79.88	SRR3066674
NSUI245	Canada	30	30	Novel	100	152	32	27	145	36	18	-	-	-	+	1,193,296	67.80	SRR3066683
NSUI246	Canada	30	30	Novel	27	77	58	129	38	34	72	-	-	-	+	1,492,878	84.82	SRR3066685
NSUI247	Canada	30	30	Novel	100	13	143	155	109	34	102	-	-	-	+	1,303,268	74.05	SRR3066686
NSUI248	Canada	30	30	Novel	31	177	181	15	10	156	145	+	-	+	+	1,201,870	68.29	SRR3066687
NSUI249	Canada	31	31	Novel	73	232	76	126	152	163	66	-	-	-	+	1,390,202	78.99	SRR3066720
NSUI250	Canada	31	31	Novel	188	10	28	148	8	10	27	-	-	-	+	946,694	53.79	SRR3066721
NSUI251	Canada	31	31	Novel	119	88	30	73	139	20	33	-	-	-	+	1,082,324	61.50	SRR3066722
NSUI255	Canada	9	9	Novel	17	33	53	30	2	6	8	-	-	-	+	1,372,842	78.00	SRR3066723
NSUI256	Canada	9	9	Novel	17	68	21	4	14	6	8	+	-	+	+	1,042,146	59.21	SRR3066724
NSUI257	Canada	9	9	Novel	23	25	7	32	16	18	37	-	-	-	+	1,249,014	70.97	SRR3066725
DAN 12814	DK[g]	11	11	91	31	18	5	12	41	10	18	+	-	-	+	730,616	49.81	SRR3066726
NCTC 10446	NL[h]	15	15	81	22	1	20	21	4	21	26	+	-	+	+	667,960	45.54	SRR3066728
NSUI262	Canada	1/2	ND	28	ND[i]	ND	ND	ND	ND	ND	ND	ND	ND	ND	ND	ND	ND	ND
NSUI264	Canada	1/2	ND	28	ND	ND	ND	ND	ND	ND	ND	ND	ND	ND	ND	ND	ND	ND
NSUI266	Canada	1/2	ND	28	ND	ND	ND	ND	ND	ND	ND	ND	ND	ND	ND	ND	ND	ND

Strain	Origin	Serotype		Sequence Type[b]	*aroA*	*cpn60*	*dpr*	*gki*	*mutS*	*recA*	*thrA*	*mrp*	*epf*	*sly*	*recN*	Illumina Sequence Data		SRA Accession №
		Coag[a]	Pipeline													№ of Reads	Coverage	
NSUI267	Canada	1/2	ND	28	ND	ND	ND	ND	ND	ND	ND	ND	ND	ND	ND	ND	ND	ND
NSUI268	Canada	1/2	ND	28	ND	ND	ND	ND	ND	ND	ND	ND	ND	ND	ND	ND	ND	ND
NSUI269	Canada	1/2	ND	28	ND	ND	ND	ND	ND	ND	ND	ND	ND	ND	ND	ND	ND	ND
NSUI270	Canada	1/2	ND	28	ND	ND	ND	ND	ND	ND	ND	ND	ND	ND	ND	ND	ND	ND
NSUI271	Canada	1/2	ND	28	ND	ND	ND	ND	ND	ND	ND	ND	ND	ND	ND	ND	ND	ND
NSUI272	Canada	1/2	ND	28	ND	ND	ND	ND	ND	ND	ND	ND	ND	ND	ND	ND	ND	ND
NSUI273	Canada	1/2	ND	28	ND	ND	ND	ND	ND	ND	ND	ND	ND	ND	ND	ND	ND	ND
NSUI275	Canada	1/2	ND	28	ND	ND	ND	ND	ND	ND	ND	ND	ND	ND	ND	ND	ND	ND
NSUI276	Canada	1/2	ND	28	ND	ND	ND	ND	ND	ND	ND	ND	ND	ND	ND	ND	ND	ND
NSUI277	Canada	1/2	ND	28	ND	ND	ND	ND	ND	ND	ND	ND	ND	ND	ND	ND	ND	ND

[a] Coag: Coagglutination

[b] Sequence type based on SRST2 MLST results

[c] Novel: Allele or allele combinations not found in MLST database

[d] NT: Non-typeable. This strain was re-sequenced and serotype was assigned by the pipeline as result shown in brackets.

[e] LC: Low Coverage; the coverage was too low to call an allele by the program SRST2, but manual inspection of the BAM files revealed a result shown in brackets.

[f] SRA accession of the re-sequenced strain is SRR3458042.

[g] DK: Denmark.

[h] NL: The Netherlands.

[i] ND: Not done.

Table S2. Serotypes and accession numbers for reference capsule type sequences used in this study.

Serotypes	GenBank accession no.
**1**	JF273644
**2**	AF118389
**3**	JF273646
**4**	JF273647
**5**	JF273648
**6**	AB737818
**7**	JF273649
**8**	JF273650
**9**	JF273651
**10**	JF273652
**11**	AB737819
**12**	AB737820
**13**	AB737821
**15**	AB737823
**16**	HQ694980
**17**	AB737824
**18**	AB737825
**19**	JF273654
**21**	AB737827
**23**	JF273655
**24**	AB737829
**25**	JF273656
**27**	AB737831
**28**	AB737832
**29**	AB737833
**30**	AB737834
**31**	AB737835

Table S3. Information and pipeline results of *S. suis* strains downloaded from the Sequence Read Archive

Strain	Serotype		Source	Host	Accession Number
	Published	Pipeline			
S11H	1	1	UK	Pig	ERS132481
S13C	1	1	UK	Pig	ERS132494
S13H	1	1	UK	Pig	ERS132497
LL-Q	1	1	UK	Pig	ERS132353
LL-R	1	1	UK	Pig	ERS132354
S13I	1	1	UK	Pig	ERS132498
S93M	1	1	UK	Pig	ERS132524
S97P	1	1	UK	Pig	ERS132527
S11J	1	-[a]	UK	Pig	ERS132539
S11K	1	3	UK	Pig	ERS132540
S14M	1	1/2	UK	Pig	ERS132548
LS8X	1	12	UK	Pig	ERS132440
LS5Q	1	11	UK	Pig	ERS132457
LS6R	1	11	UK	Pig	ERS132458
LS0C	1	15	UK	Pig	ERS132462
LS9U	1	30	UK	Pig	ERS132461
LSW	1	15	UK	Pig	ERS132364
S15Y	1	1	UK	Pig	ERS132511
LL-P	2	2	UK	Pig	ERS132352
S10Y	2	2	UK	Pig	ERS132474
S10E	2	2	UK	Pig	ERS132538
S11I	2	2	UK	Pig	ERS132482
S12T	2	2	UK	Pig	ERS132487
S12V	2	2	UK	Pig	ERS132489
S15T	2	2	UK	Pig	ERS132506
S15V	2	2	UK	Pig	ERS132508
S16B	2	2	UK	Pig	ERS132512
S16D	2	2	UK	Pig	ERS132514
S16E	2	2	UK	Pig	ERS132515
S17G	2	2	UK	Pig	ERS132517
S17H	2	2	UK	Pig	ERS132518
S90C	2	2	UK	Pig	ERS132464
S92D	2	2	UK	Pig	ERS132465
S93E	2	2	UK	Pig	ERS132466
S96H	2	2	UK	Pig	ERS132468
S91R	2	2	UK	Pig	ERS132529
S94U	2	2	UK	Pig	ERS132532

Strain	Serotype		Source	Host	Accession Number
	Published	Pipeline			
S10X	2	2	UK	Pig	ERS132473
S10A	2	2	UK	Pig	ERS132475
S11G	2	2	UK	Pig	ERS132480
S12S	2	2	UK	Pig	ERS132486
S13B	2	2	UK	Pig	ERS132493
S13F	2	2	UK	Pig	ERS132496
S15U	2	2	UK	Pig	ERS132507
S94F	2	2	UK	Pig	ERS132467
S11N	2	2	UK	Pig	ERS132485
S12U	2	2	UK	Pig	ERS132488
S13D	2	2	UK	Pig	ERS132495
S16C	2	2	UK	Pig	ERS132513
S99B	2	2	UK	Pig	ERS132463
S99Q	2	2	UK	Pig	ERS132528
S92S	2	2	UK	Pig	ERS132530
S98Y	2	2	UK	Pig	ERS132471
LSL	2	2	UK	Pig	ERS132363
LS0E	2	31	UK	Pig	ERS132422
LS1F	2	1/2	UK	Pig	ERS132423
LS2G	2	2	UK	Pig	ERS132424
LS3H	2	2	UK	Pig	ERS132425
LS1Q	2	1/2	UK	Pig	ERS132433
LS2R	2	1/2	UK	Pig	ERS132434
LS8U	2	2	UK	Pig	ERS132390
LSS	2	2	UK	Pig	ERS132366
LSD	2	2	UK	Pig	ERS132367
LS1N	2	2	UK	Pig	ERS132383
LS2O	2	2	UK	Pig	ERS132384
LS5C	2	2	UK	Pig	ERS132397
LS0T	2	2	UK	Pig	ERS132412
LS1U	2	2	UK	Pig	ERS132413
LS7S	2	2	UK	Pig	ERS132459
LS8T	2	2	UK	Pig	ERS132460
S10C	2	2	UK	Pig	ERS132477
B18E	2	2	Vietnam	Human	ERS156197
B10F	2	2	Vietnam	Human	ERS156198
B11G	2	2	Vietnam	Human	ERS156199
B17H	2	2	Vietnam	Human	ERS156200
B18I	2	2	Vietnam	Human	ERS156201
B23J	2	2	Vietnam	Human	ERS156202

Strain	Serotype		Source	Host	Accession Number
	Published	Pipeline			
B29K	2	2	Vietnam	Human	ERS156203
B21L	2	2	Vietnam	Human	ERS156204
B23M	2	2	Vietnam	Human	ERS156205
B26N	2	2	Vietnam	Human	ERS156206
B24O	2	2	Vietnam	Human	ERS156207
B28P	2	2	Vietnam	Human	ERS156208
B21Q	2	2	Vietnam	Human	ERS156209
B27R	2	2	Vietnam	Human	ERS156210
B21S	2	2	Vietnam	Human	ERS156211
B21T	2	2	Vietnam	Human	ERS156212
B23U	2	2	Vietnam	Human	ERS156213
B27V	2	2	Vietnam	Human	ERS156214
B24W	2	2	Vietnam	Human	ERS156215
B22X	2	2	Vietnam	Human	ERS156216
B28Y	2	2	Vietnam	Human	ERS156217
B33Z	2	2	Vietnam	Human	ERS156218
B38A	2	2	Vietnam	Human	ERS018463
B35B	2	2	Vietnam	Human	ERS156220
B31C	2	2	Vietnam	Human	ERS156221
B39D	2	2	Vietnam	Human	ERS156222
B34E	2	2	Vietnam	Human	ERS156223
B35F	2	2	Vietnam	Human	ERS156224
B36G	2	2	Vietnam	Human	ERS156225
B37H	2	2	Vietnam	Human	ERS156226
B38I	2	2	Vietnam	Human	ERS156227
B32J	2	2	Vietnam	Human	ERS156228
B36K	2	2	Vietnam	Human	ERS156229
B33L	2	2	Vietnam	Human	ERS156230
B41M	2	2	Vietnam	Human	ERS156231
B48N	2	2	Vietnam	Human	ERS156232
B44O	2	2	Vietnam	Human	ERS156233
B46P	2	2	Vietnam	Human	ERS156234
B41Q	2	2	Vietnam	Human	ERS156235
B44R	2	2	Vietnam	Human	ERS156236
B42S	2	2	Vietnam	Human	ERS156237
B40T	2	2	Vietnam	Human	ERS156238
B41U	2	2	Vietnam	Human	ERS156239
B45V	2	2	Vietnam	Human	ERS156240
B46W	2	2	Vietnam	Human	ERS156241
B41X	2	2	Vietnam	Human	ERS156242
B48Y	2	2	Vietnam	Human	ERS156243

Strain	Serotype		Source	Host	Accession Number
	Published	Pipeline			
E03B	2	2	Vietnam	Human	ERS156244
E00C	2	2	Vietnam	Human	ERS156245
E05E	2	2	Vietnam	Human	ERS156246
E00F	2	2	Vietnam	Human	ERS156247
E03G	2	2	Vietnam	Human	ERS156248
E04H	2	2	Vietnam	Human	ERS156249
E01I	2	2	Vietnam	Human	ERS156250
E04J	2	2	Vietnam	Human	ERS156251
E00K	2	2	Vietnam	Human	ERS156252
E08L	2	2	Vietnam	Human	ERS156253
E01D	2	2	Vietnam	Human	ERS156254
E14M	2	2	Vietnam	Human	ERS156255
E10N	2	2	Vietnam	Human	ERS156256
E11O	2	2	Vietnam	Human	ERS156257
E17P	2	2	Vietnam	Human	ERS156258
E11Q	2	14	Vietnam	Human	ERS156259
E21R	2	2	Vietnam	Human	ERS156260
E23S	2	2	Vietnam	Human	ERS156261
E24T	2	2	Vietnam	Human	ERS156262
E21U	2	2	Vietnam	Human	ERS156263
E21V	2	2	Vietnam	Human	ERS156264
E34W	2	14	Vietnam	Human	ERS156265
E36X	2	2	Vietnam	Human	ERS156266
E30Y	2	2	Vietnam	Human	ERS156267
E30Z	2	2	Vietnam	Human	ERS156268
E39A	2	2	Vietnam	Human	ERS156269
E56B	2	2	Vietnam	Human	ERS156270
E57C	2	2	Vietnam	Human	ERS156271
E58D	2	2	Vietnam	Human	ERS156272
E55E	2	2	Vietnam	Human	ERS156273
E63F	2	2	Vietnam	Human	ERS156274
E66G	2	2	Vietnam	Human	ERS156275
E62H	2	2	Vietnam	Human	ERS156276
E60I	2	2	Vietnam	Human	ERS156277
S1J	2	2	Vietnam	Human	ERS156311
S1K	2	2	Vietnam	Human	ERS156312
S1L	2	2	Vietnam	Human	ERS156313
S1M	2	2	Vietnam	Human	ERS156314
S1N	2	2	Vietnam	Human	ERS156315
S1O	2	2	Vietnam	Human	ERS156316
S2P	2	2	Vietnam	Human	ERS156317

Strain	Serotype		Source	Host	Accession Number
	Published	Pipeline			
S2Q	2	2	Vietnam	Human	ERS156318
S2R	2	2	Vietnam	Human	ERS156319
S2S	2	2	Vietnam	Human	ERS156320
S2T	2	2	Vietnam	Human	ERS156321
S2U	2	2	Vietnam	Human	ERS156322
S2V	2	2	Vietnam	Human	ERS156323
S2W	2	2	Vietnam	Human	ERS156324
S2X	2	2	Vietnam	Human	ERS156325
S2Y	2	2	Vietnam	Human	ERS156326
S3Z	2	2	Vietnam	Human	ERS156327
S3A	2	2	Vietnam	Human	ERS156328
S3B	2	2	Vietnam	Human	ERS156329
S3C	2	2	Vietnam	Human	ERS156330
S3D	2	2	Vietnam	Human	ERS156331
S3E	2	2	Vietnam	Human	ERS156332
S3F	2	2	Vietnam	Human	ERS156333
S3G	2	2	Vietnam	Human	ERS156334
S4H	2	2	Vietnam	Human	ERS156335
S4I	2	2	Vietnam	Human	ERS156336
S4J	2	2	Vietnam	Human	ERS156337
S4K	2	2	Vietnam	Human	ERS156338
S6L	2	2	Vietnam	Human	ERS156339
S6M	2	2	Vietnam	Human	ERS156340
S6N	2	-	Vietnam	Human	ERS156341
S6O	2	2	Vietnam	Human	ERS156342
S7P	2	2	Vietnam	Human	ERS156343
S7Q	2	2	Vietnam	Human	ERS156344
S7R	2	2	Vietnam	Human	ERS156345
S7S	2	2	Vietnam	Human	ERS156346
S7T	2	2	Vietnam	Human	ERS156347
S8U	2	2	Vietnam	Human	ERS156348
S8V	2	2	Vietnam	Human	ERS156349
S8W	2	2	Vietnam	Human	ERS156350
S8X	2	2	Vietnam	Human	ERS156351
S8Y	2	2	Vietnam	Human	ERS156352
S8Z	2	2	Vietnam	Human	ERS156353
S9J	2	2	Vietnam	Human	ERS156354
S9A	2	2	Vietnam	Human	ERS156355
S11P	2	2	Vietnam	Human	ERS156356
S12Z	2	2	Vietnam	Human	ERS156357
V20B	2	2	Vietnam	Human	ERS156358

Strain	Serotype		Source	Host	Accession Number
	Published	Pipeline			
V22C	2	2	Vietnam	Human	ERS156359
V27D	2	2	Vietnam	Human	ERS156360
V32E	2	2	Vietnam	Human	ERS156361
V39F	2	2	Vietnam	Human	ERS156362
V30G	2	2	Vietnam	Human	ERS156363
V36H	2	2	Vietnam	Human	ERS156364
V31I	2	2	Vietnam	Human	ERS156365
V31J	2	2	Vietnam	Human	ERS156366
V33K	2	2	Vietnam	Human	ERS156367
V34L	2	2	Vietnam	Human	ERS156368
V36M	2	2	Vietnam	Human	ERS156369
V37N	2	2	Vietnam	Human	ERS156370
V38O	2	2	Vietnam	Human	ERS156371
V34P	2	2	Vietnam	Human	ERS156372
V39Q	2	2	Vietnam	Human	ERS156373
V30R	2	2	Vietnam	Human	ERS156374
V35S	2	2	Vietnam	Human	ERS156375
V30T	2	2	Vietnam	Human	ERS156376
V36U	2	2	Vietnam	Human	ERS156377
V36V	2	2	Vietnam	Human	ERS156378
V37W	2	2	Vietnam	Human	ERS156379
V38X	2	2	Vietnam	Human	ERS156380
V38Y	2	2	Vietnam	Human	ERS156381
V38Z	2	2	Vietnam	Human	ERS156382
0Z-A	2	2	Vietnam	Pig	ERS156386
0Z-B	2	2	Vietnam	Pig	ERS156383
0Z-C	2	2	Vietnam	Pig	ERS156384
0Z-D	2	2	Vietnam	Pig	ERS156385
CU2A	2	2	Vietnam	Pig	ERS156388
CU2Z	2	2	Vietnam	Pig	ERS156387
F1L	2	2	Vietnam	Pig	ERS156279
F1N	2	2	Vietnam	Pig	ERS156280
F2P	2	2	Vietnam	Pig	ERS156281
F2S	2	2	Vietnam	Pig	ERS156282
F4V	2	2	Vietnam	Pig	ERS156283
F5D	2	2	Vietnam	Pig	ERS156284
F5F	2	2	Vietnam	Pig	ERS156285
F5K	2	2	Vietnam	Pig	ERS156286
F7L	2	2	Vietnam	Pig	ERS156287
F7M	2	2	Vietnam	Pig	ERS156288

Strain	Serotype		Source	Host	Accession Number
	Published	Pipeline			
F7N	2	2	Vietnam	Pig	ERS156289
F9O	2	2	Vietnam	Pig	ERS156290
F19J	2	2	Vietnam	Pig	ERS156278
F17K	2	2	Vietnam	Pig	ERS156292
F14M	2	2	Vietnam	Pig	ERS156293
F21O	2	2	Vietnam	Pig	ERS156294
F20Q	2	2	Vietnam	Pig	ERS156295
F25R	2	2	Vietnam	Pig	ERS156296
F38T	2	2	Vietnam	Pig	ERS156297
F36U	2	2	Vietnam	Pig	ERS156298
F45W	2	2	Vietnam	Pig	ERS156299
F49X	2	2	Vietnam	Pig	ERS156300
F48Y	2	2	Vietnam	Pig	ERS156301
F41Z	2	2	Vietnam	Pig	ERS156302
F48A	2	2	Vietnam	Pig	ERS156303
F43B	2	2	Vietnam	Pig	ERS156304
F51C	2	2	Vietnam	Pig	ERS156305
F57E	2	2	Vietnam	Pig	ERS156306
F57G	2	2	Vietnam	Pig	ERS156307
F55H	2	2	Vietnam	Pig	ERS156308
F50I	2	2	Vietnam	Pig	ERS156309
F53J	2	2	Vietnam	Pig	ERS156310
LL-T	3	3	UK	Pig	ERS132356
LL-S	3	3	UK	Pig	ERS132355
S10B	3	3	UK	Pig	ERS132476
S11L	3	3	UK	Pig	ERS132483
S11M	3	8	UK	Pig	ERS132484
S12Q	3	3	UK	Pig	ERS132542
S15S	3	3	UK	Pig	ERS132549
S15X	3	3	UK	Pig	ERS132510
S99Z	3	3	UK	Pig	ERS132536
LS7T	3	10	UK	Pig	ERS132389
LS3S	3	3	UK	Pig	ERS132435
LS4T	3	3	UK	Pig	ERS132436
S13A	3	3	UK	Pig	ERS132492
S12X	4	4	UK	Pig	ERS132544
LL-U	4	4	UK	Pig	ERS132357
S14J	4	4	UK	Pig	ERS132547
S14K	4	4	UK	Pig	ERS132499
S14L	4	4	UK	Pig	ERS132500
S14O	4	4	UK	Pig	ERS132502

Strain	Serotype		Source	Host	Accession Number
	Published	Pipeline			
LS0M	4	4	UK	Pig	ERS132382
LS7A	4	-	UK	Pig	ERS132419
LS4P	4	4	UK	Pig	ERS132456
S97A	4	4	UK	Pig	ERS132520
LS3A	4	4	UK	Pig	ERS132395
LS1M	6	6	UK	Pig	ERS132453
LL-X	7	7	UK	Pig	ERS132360
S10D	7	7	UK	Pig	ERS132478
S15R	7	7	UK	Pig	ERS132505
LL-W	7	7	UK	Pig	ERS132359
S10W	7	7	UK	Pig	ERS132472
S11O	7	7	UK	Pig	ERS132541
S12R	7	7	UK	Pig	ERS132543
LS3D	7	7	UK	Pig	ERS132445
LS4E	7	7	UK	Pig	ERS132446
LS5F	7	7	UK	Pig	ERS132447
LS6Z	7	7	UK	Pig	ERS132418
S13E	8	8	UK	Pig	ERS132545
S13G	8	8	UK	Pig	ERS132546
S16Z	8	8	UK	Pig	ERS132550
S91K	9	9	UK	Pig	ERS132522
S15W	9	8	UK	Pig	ERS132509
S96W	9	1/2	UK	Pig	ERS132534
S97X	9	8	UK	Pig	ERS132535
S96O	12	12	UK	Pig	ERS132526
LL-Z	14	14	UK	Pig	ERS132362
LL-Y	14	14	UK	Pig	ERS132361
S12Y	14	14	UK	Pig	ERS132491
S99I	14	14	UK	Pig	ERS132469
S92L	14	14	UK	Pig	ERS132523
S95V	14	14	UK	Pig	ERS132533
LS2D	14	25	UK	Pig	ERS132374
LS3E	14	16	UK	Pig	ERS132375
LS4F	14	14	UK	Pig	ERS132376
S12W	14	14	UK	Pig	ERS132490
S16F	16	16	UK	Pig	ERS132516
S95G	23	-	UK	Pig	ERS132521
S17I	1/2	1/2	UK	Pig	ERS132519
S10F	1/2	1/2	UK	Pig	ERS132479
S16A	1/2	15	UK	Pig	ERS132551
LS7W	1/2	1/2	UK	Pig	ERS132439

Strain	Serotype		Source	Host	Accession Number
	Published	Pipeline			
LS5R	1/2	1/2	UK	Pig	ERS132387
LSH	1/2	1/2	UK	Pig	ERS132365
LS6G	1/2	1/2	UK	Pig	ERS132448
LS4Q	1/2	1/2	UK	Pig	ERS132386
LS6S	1/2	1/2	UK	Pig	ERS132388
LS9V	1/2	1/2	UK	Pig	ERS132391
LS7H	1/2	1/2	UK	Pig	ERS132449
LS1C	Non-typeable	31	UK	Pig	ERS132373
LS8J	Non-typeable	16	UK	Pig	ERS132380
LS3P	Non-typeable	24	UK	Pig	ERS132385
LS0X	Non-typeable	31	UK	Pig	ERS132392
LS1Y	Non-typeable	24	UK	Pig	ERS132393
LS2Z	Non-typeable	24	UK	Pig	ERS132394
LS4B	Non-typeable	8	UK	Pig	ERS132396
LS7E	Non-typeable	24	UK	Pig	ERS132399
LS8F	Non-typeable	-	UK	Pig	ERS132400
LS9G	Non-typeable	31	UK	Pig	ERS132401
LS0I	Non-typeable	6	UK	Pig	ERS132402
LS1J	Non-typeable	21	UK	Pig	ERS132403
LS2K	Non-typeable	16	UK	Pig	ERS132404
LS4M	Non-typeable	15	UK	Pig	ERS132406
LS7P	Non-typeable	16	UK	Pig	ERS132409
LS8Q	Non-typeable	31	UK	Pig	ERS132410
LS9R	Non-typeable	-	UK	Pig	ERS132411
LS3W	Non-typeable	21	UK	Pig	ERS132415
LS4X	Non-typeable	-	UK	Pig	ERS132416
LS5Y	Non-typeable	9	UK	Pig	ERS132417
LS8B	Non-typeable	31	UK	Pig	ERS132420
LS9C	Non-typeable	10	UK	Pig	ERS132421
LS4I	Non-typeable	21	UK	Pig	ERS132426
LS5J	Non-typeable	10	UK	Pig	ERS132427
LS7L	Non-typeable	8	UK	Pig	ERS132429
LS8M	Non-typeable	8	UK	Pig	ERS132430
LS9N	Non-typeable	31	UK	Pig	ERS132431
LS0P	Non-typeable	9	UK	Pig	ERS132432
LS5U	Non-typeable	31	UK	Pig	ERS132437
LS6V	Non-typeable	8	UK	Pig	ERS132438
LS9Y	Non-typeable	15	UK	Pig	ERS132441
LS0A	Non-typeable	31	UK	Pig	ERS132442
LS1B	Non-typeable	15	UK	Pig	ERS132443
LS2C	Non-typeable	16	UK	Pig	ERS132444

**Strain**	**Serotype**		**Source**	**Host**	**Accession Number**
	Published	Pipeline			
LS8I	Non-typeable	-	UK	Pig	ERS132450
LS9J	Non-typeable	8	UK	Pig	ERS132451
LSV	Non-typeable	10	UK	Pig	ERS132371
LS0L	Non-typeable	31	UK	Pig	ERS132452
LS2N	Non-typeable	9	UK	Pig	ERS132454
S93T	Non-typeable	-	UK	Pig	ERS132531
LS0B	Non-typeable	-	UK	Pig	ERS132372
LSK	Non-typeable	16	UK	Pig	ERS132370
S95N	Non-typeable	-	UK	Pig	ERS132525
LS6D	Non-typeable	-	UK	Pig	ERS132398
LS2V	Non-typeable	19	UK	Pig	ERS132414
LS6K	Non-typeable	9	UK	Pig	ERS132428

[a] -: No serotype was called for this strain, indicating that it is a non-typeable

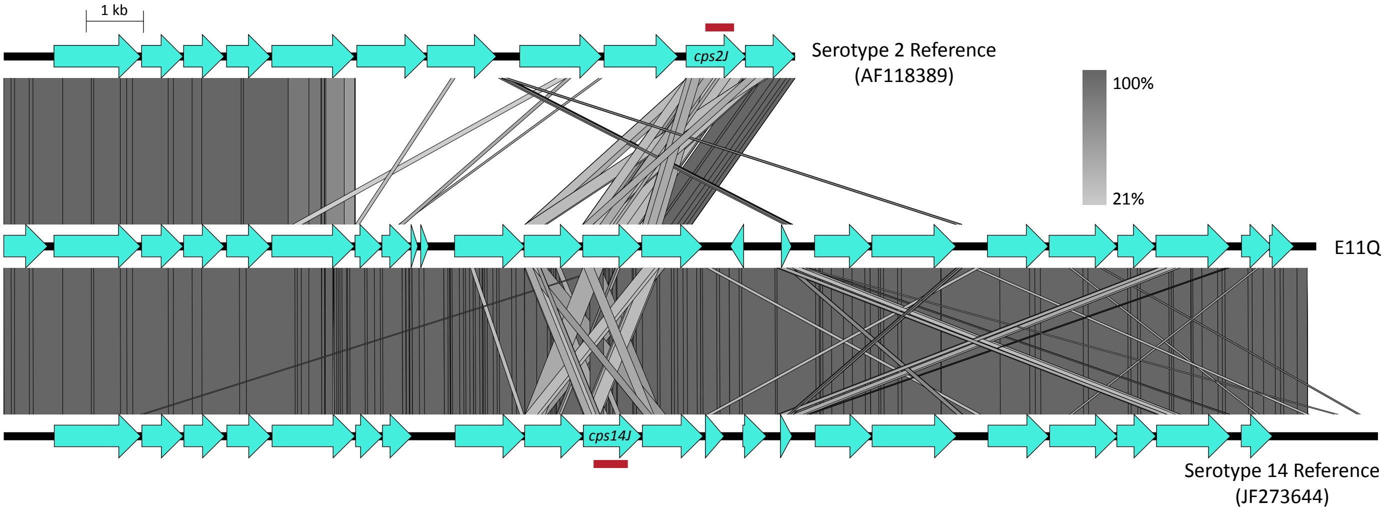

Figure S1. Comparison of *cps* loci show that strain E11Q is actually serotype 14, as identified by our pipeline. Strain W11Q had been typed as serotype 2 by serological methods. Short-read WGS data for strain E11Q were downloaded from the Sequence Read Archive and *de novo* assembled using the A5 pipeline. The *cps* loci of a serotype 2 reference strain (top), strain E11Q (middle) and a serotype 14 reference strain (bottom) were compared using the program easyFig. Serotype specific regions searched for by our pipeline are indicated in red. GenBank Accession numbers for reference sequences are provided.

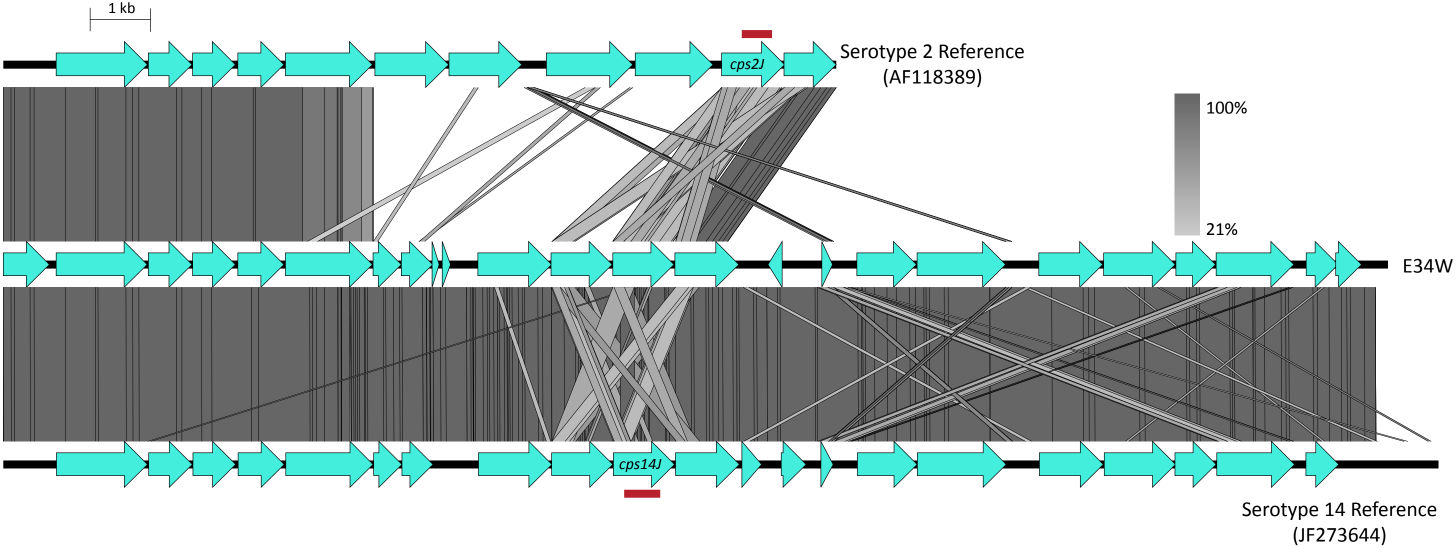

Figure S2. Comparison of *cps* loci show that strain E34W is actually serotype 14, as identified by our pipeline. Strain W11Q had been typed as serotype 2 by serological methods. Short-read WGS data for strain E34W were downloaded from the Sequence Read Archive and *de novo* assembled using the A5 pipeline. The *cps* loci of a serotype 2 reference strain (top), strain E34W (middle) and a serotype 14 reference strain (bottom) were compared using the program easyFig. Serotype specific regions searched for by our pipeline are indicated in red. GenBank Accession numbers for reference sequences are provided.

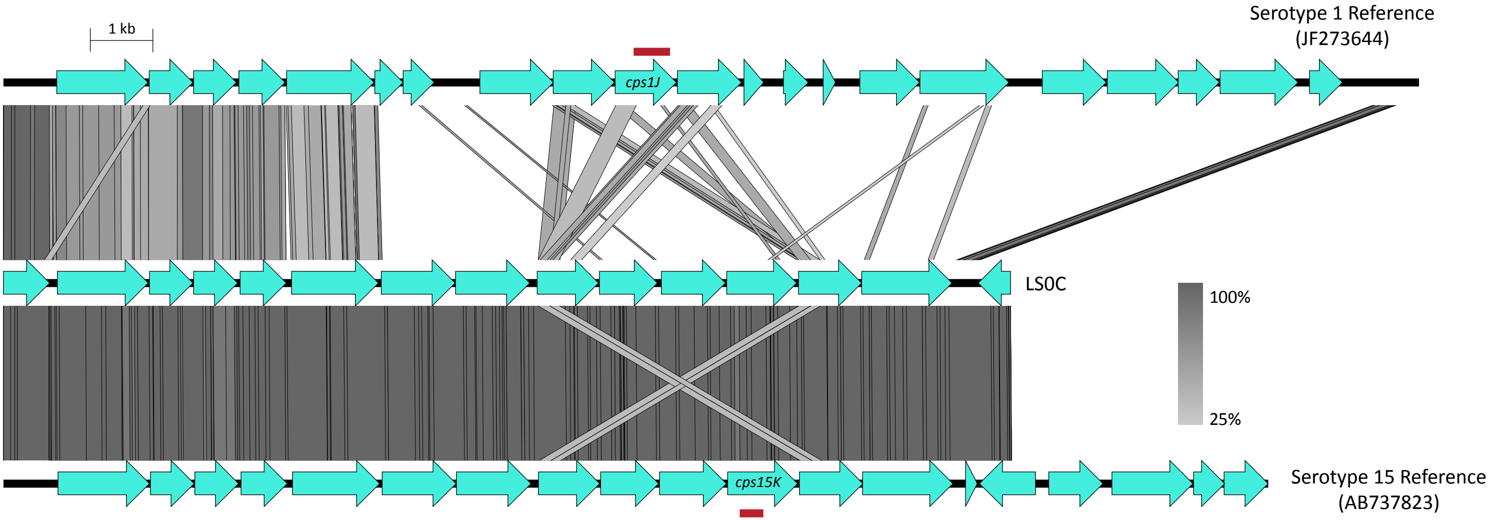

Figure S3. Comparison of *cps* loci show that strain LS0C is actually serotype 15, as identified by our pipeline. Strain LS0C had been typed as serotype 1 by serological methods. Short-read WGS data for strain LS0C were downloaded from the Sequence Read Archive and *de novo* assembled using the A5 pipeline. The *cps* loci of a serotype 1 reference strain (top), strain LS0C (middle) and a serotype 15 reference strain (bottom) were compared using the program easyFig. Serotype specific regions searched for by our pipeline are indicated in red. GenBank Accession numbers for reference sequences are provided.

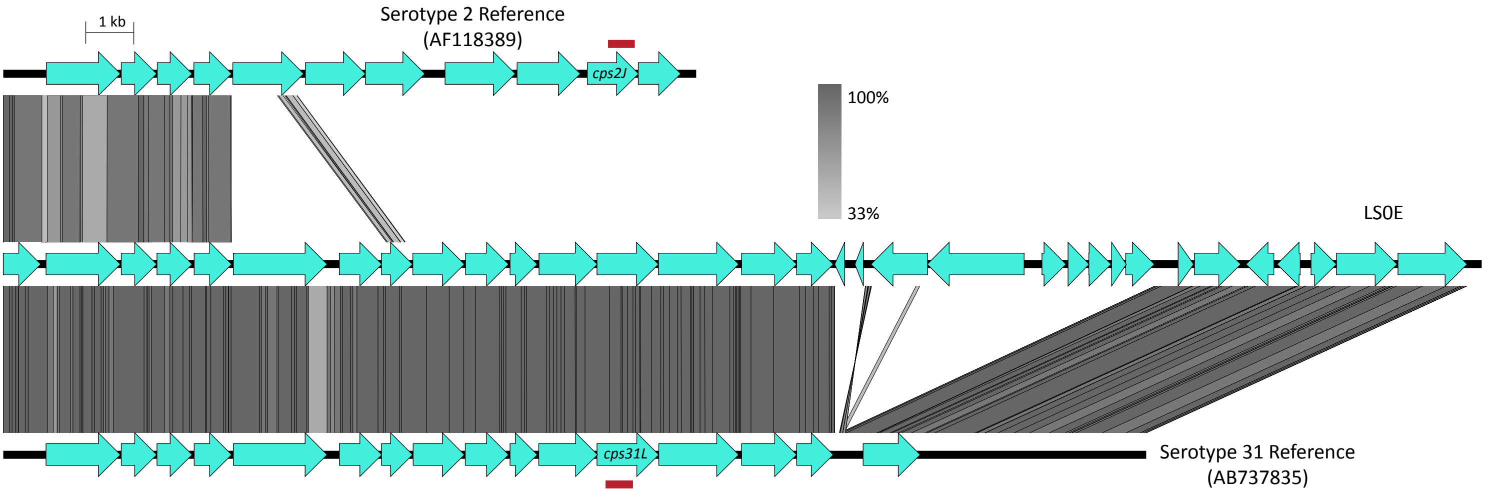

Figure S4. Comparison of *cps* loci show that strain LS0E is actually serotype 31, as identified by our pipeline. Strain LS0E had been typed as serotype 2 by serological methods. Short-read WGS data for strain LS0E were downloaded from the Sequence Read Archive and *de novo* assembled using the A5 pipeline. The *cps* loci of a serotype 2 reference strain (top), strain LS0E (middle) and a serotype 31 reference strain (bottom) were compared using the program easyFig. Serotype specific regions searched for by our pipeline are indicated in red. GenBank Accession numbers for reference sequences are provided.

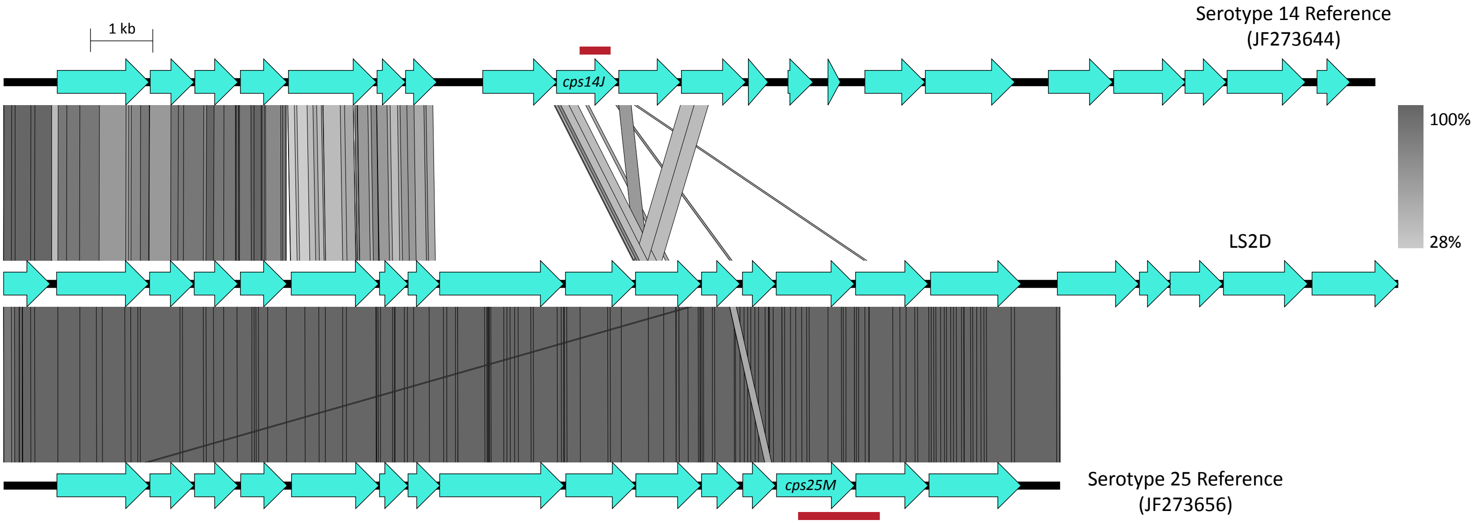

Figure S5. Comparison of *cps* loci show that strain LS2D is actually serotype 25, as identified by our pipeline. Strain LS2D had been typed as serotype 14 by serological methods. Short-read WGS data for strain LS2D were downloaded from the Sequence Read Archive and *de novo* assembled using the A5 pipeline. The *cps* loci of a serotype 14 reference strain (top), strain LS2D (middle) and a serotype 25 reference strain (bottom) were compared using the program easyFig. Serotype specific regions searched for by our pipeline are indicated in red. GenBank Accession numbers for reference sequences are provided.

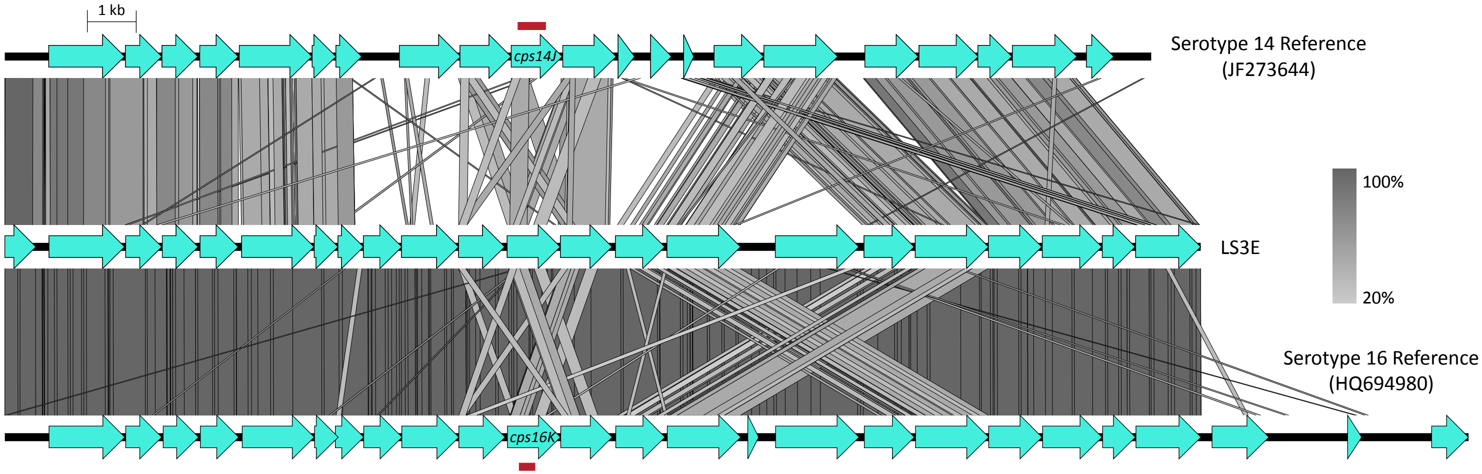

Figure S6. Comparison of *cps* loci show that strain LSE3 is actually serotype 16, as identified by our pipeline. Strain LS3E had been typed as serotype 14 by serological methods. Short-read WGS data for strain LS3E were downloaded from the Sequence Read Archive and *de novo* assembled using the A5 pipeline. The *cps* loci of a serotype 14 reference strain (top), strain LS3E (middle) and a serotype 16 reference strain (bottom) were compared using the program easyFig. Serotype specific regions searched for by our pipeline are indicated in red. GenBank Accession numbers for reference sequences are provided.

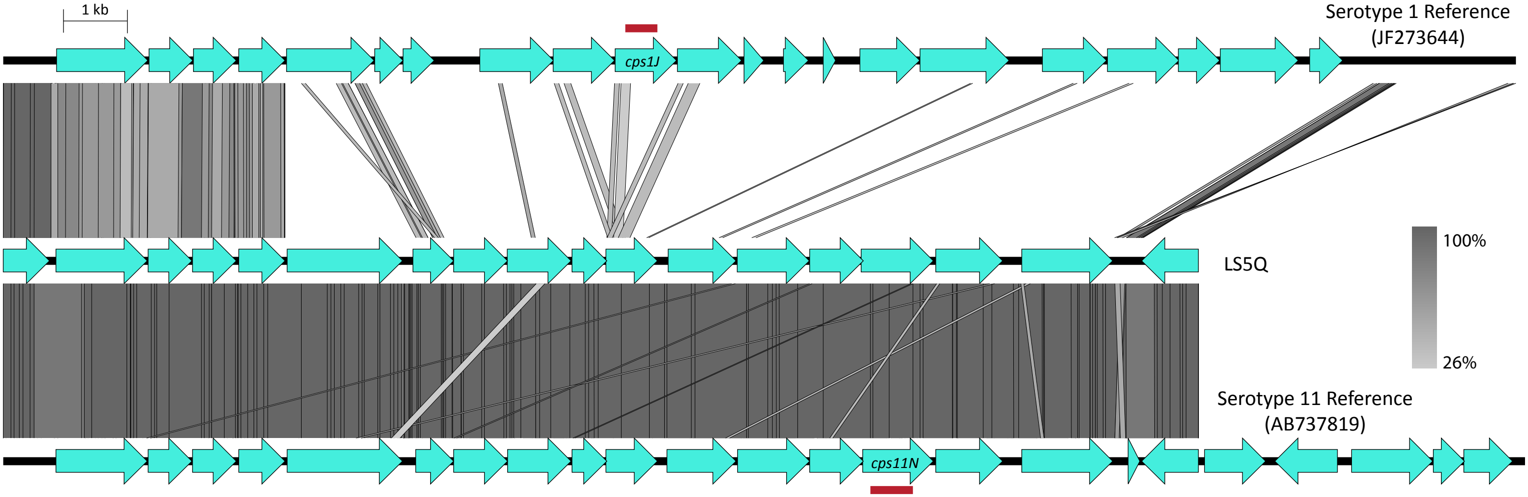

Figure S7. Comparison of *cps* loci show that strain LS5Q is actually serotype 11, as identified by our pipeline. Strain LS5Q had been typed as serotype 1 by serological methods. Short-read WGS data for strain LS5Q were downloaded from the Sequence Read Archive and *de novo* assembled using the A5 pipeline. The *cps* loci of a serotype 1 reference strain (top), strain LS5Q (middle) and a serotype 11 reference strain (bottom) were compared using the program easyFig. Serotype specific regions searched for by our pipeline are indicated in red. GenBank Accession numbers for reference sequences are provided.

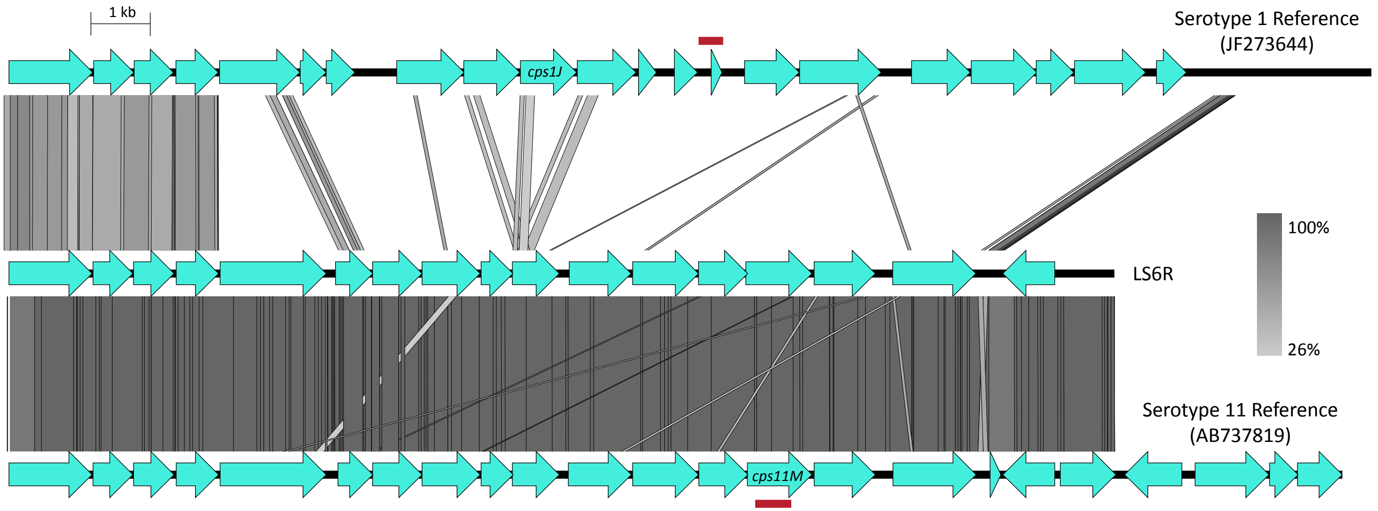

Figure S8. Comparison of *cps* loci show that strain LS6R is actually serotype 11, as identified by our pipeline. Strain LS6R had been typed as serotype 1 by serological methods. Short-read WGS data for strain LS6R were downloaded from the Sequence Read Archive and *de novo* assembled using the A5 pipeline. The *cps* loci of a serotype 1 reference strain (top), strain LS6R (middle) and a serotype 11 reference strain (bottom) were compared using the program easyFig. Serotype specific regions searched for by our pipeline are indicated in red. GenBank Accession numbers for reference sequences are provided.

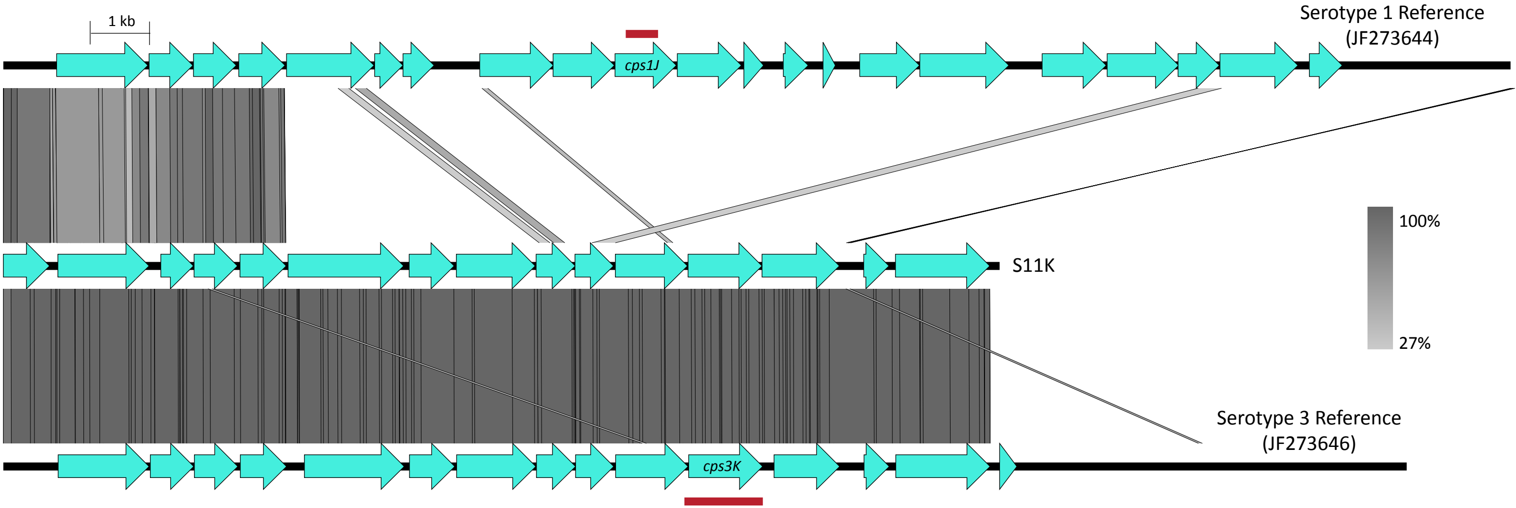

Figure S9. Comparison of *cps* loci show that strain S11K is actually serotype 3, as identified by our pipeline. Strain S11K had been typed as serotype 1 by serological methods. Short-read WGS data for strain LSW were downloaded from the Sequence Read Archive and *de novo* assembled using the A5 pipeline. The *cps* loci of a serotype 1 reference strain (top), strain S11K (middle) and a serotype 3 reference strain (bottom) were compared using the program easyFig. Serotype specific regions searched for by our pipeline are indicated in red. GenBank Accession numbers for reference sequences are provided.

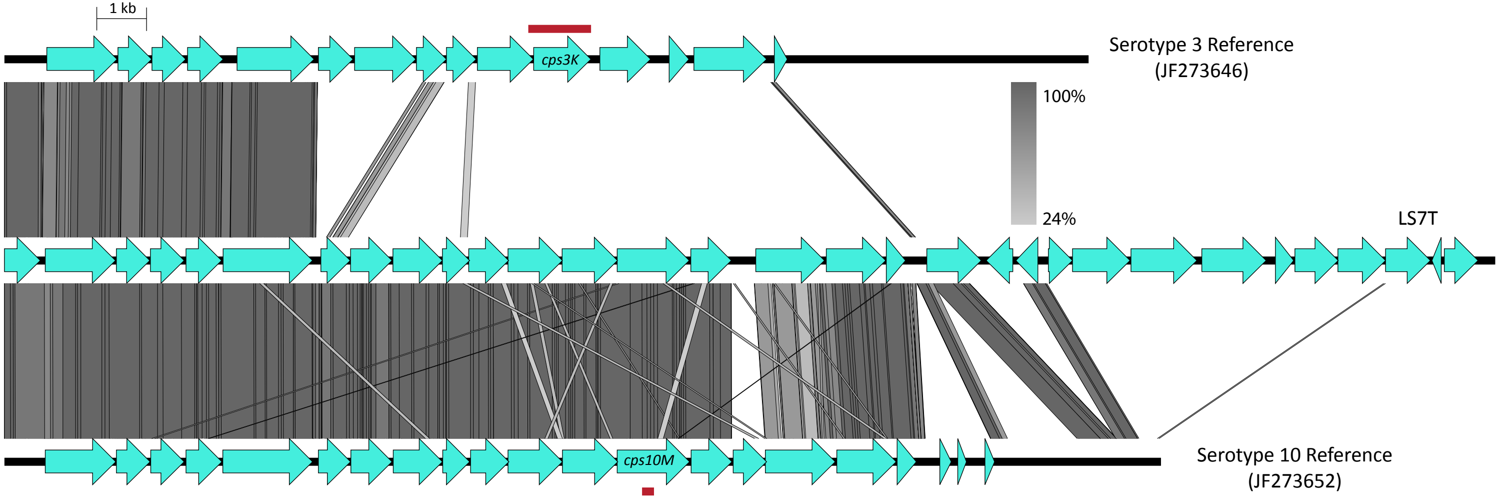

Figure S10. Comparison of *cps* loci show that strain LS7T is actually serotype 10, as identified by our pipeline. Strain LS7T had been typed as serotype 3 by serological methods. Short-read WGS data for strain LS7T were downloaded from the Sequence Read Archive and *de novo* assembled using the A5 pipeline. The *cps* loci of a serotype 3 reference strain (top), strain LS7T (middle) and a serotype 10 reference strain (bottom) were compared using the program easyFig. Serotype specific regions searched for by our pipeline are indicated in red. GenBank Accession numbers for reference sequences are provided.

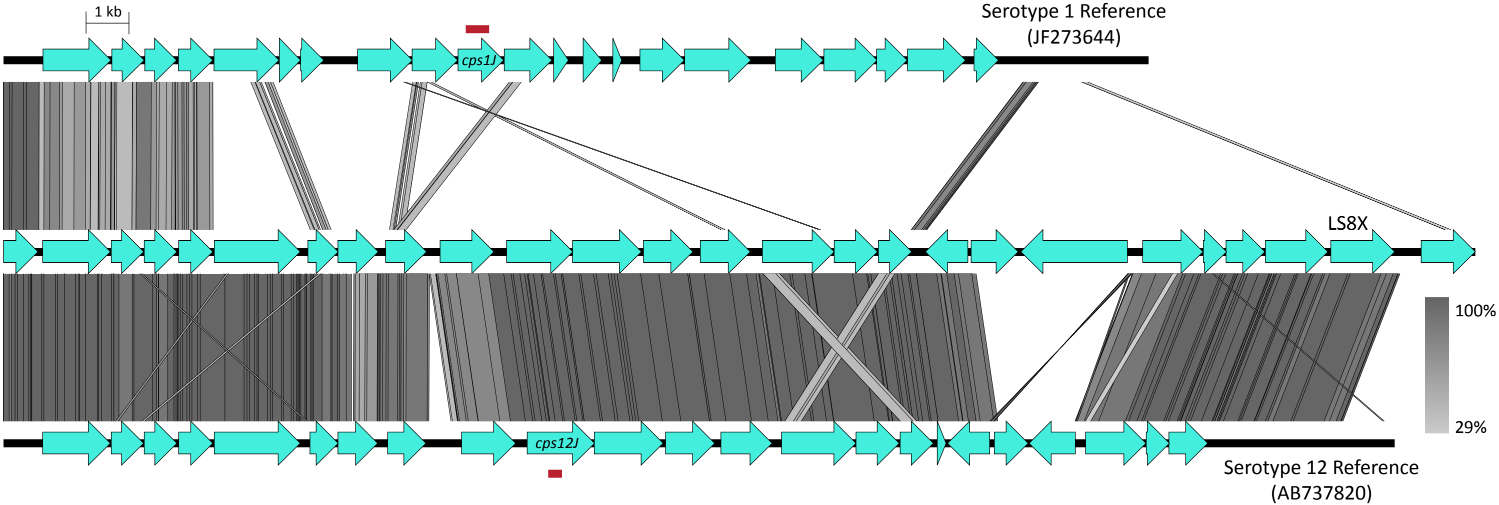

Figure S11. Comparison of *cps* loci show that strain LS8X is actually serotype 12, as identified by our pipeline. Strain LS8X had been typed as serotype 1 by serological methods. Short-read WGS data for strain LS8X were downloaded from the Sequence Read Archive and *de novo* assembled using the A5 pipeline. The *cps* loci of a serotype 1 reference strain (top), strain LS8X (middle) and a serotype 12 reference strain (bottom) were compared using the program easyFig. Serotype specific regions searched for by our pipeline are indicated in red. GenBank Accession numbers for reference sequences are provided.

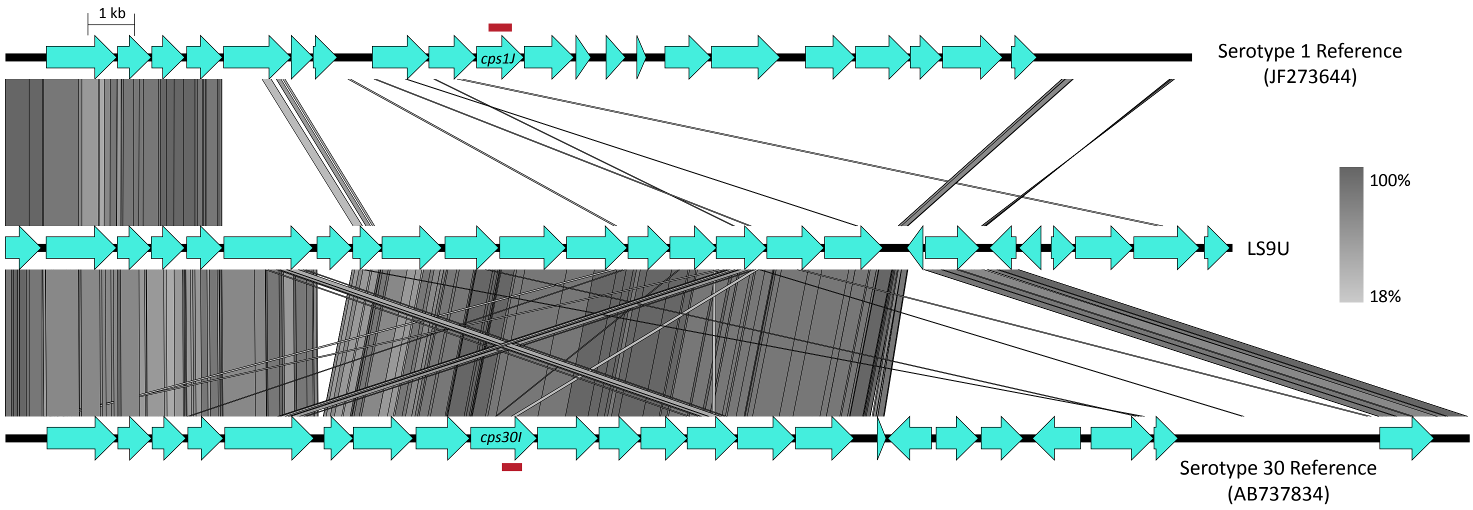

Figure S12. Comparison of *cps* loci show that strain LS9U is actually serotype 30, as identified by our pipeline. Strain LS9U had been typed as serotype 1 by serological methods. Short-read WGS data for strain LS9U were downloaded from the Sequence Read Archive and *de novo* assembled using the A5 pipeline. The *cps* loci of a serotype 1 reference strain (top), strain LS9U (middle) and a serotype 30 reference strain (bottom) were compared using the program easyFig. Serotype specific regions searched for by our pipeline are indicated in red. GenBank Accession numbers for reference sequences are provided.

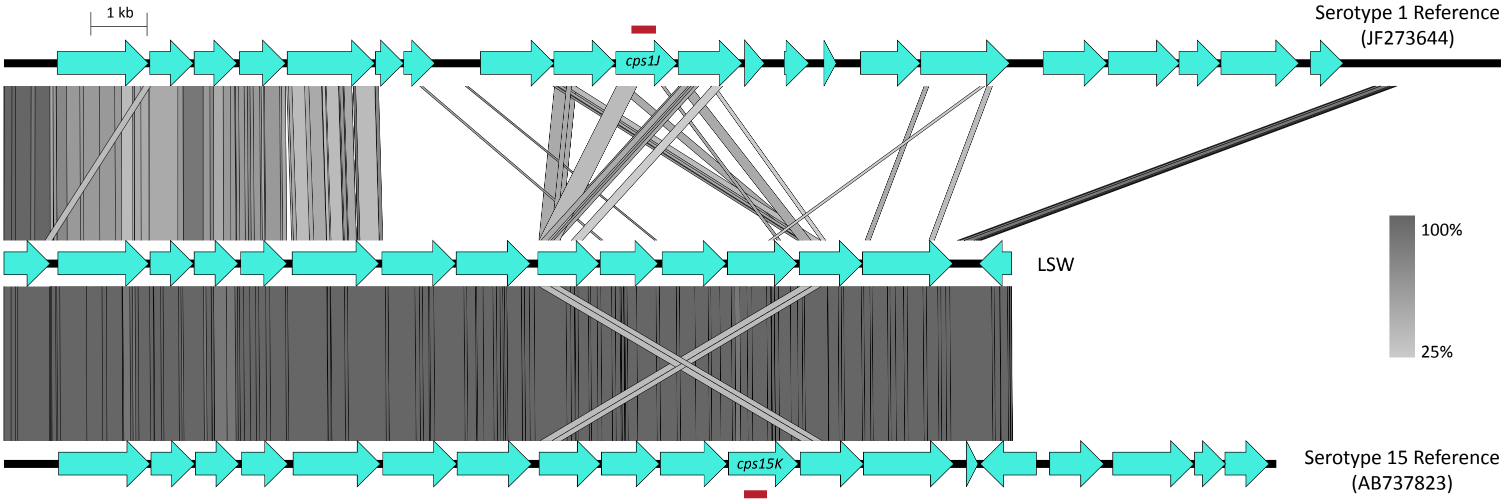

Figure S13. Comparison of *cps* loci show that strain LSW is actually serotype 15, as identified by our pipeline. Strain LSW had been typed as serotype 1 by serological methods. Short-read WGS data for strain LSW were downloaded from the Sequence Read Archive and *de novo* assembled using the A5 pipeline. The *cps* loci of a serotype 1 reference strain (top), strain LSW (middle) and a serotype 15 reference strain (bottom) were compared using the program easyFig. Serotype specific regions searched for by our pipeline are indicated in red. GenBank Accession numbers for reference sequences are provided.

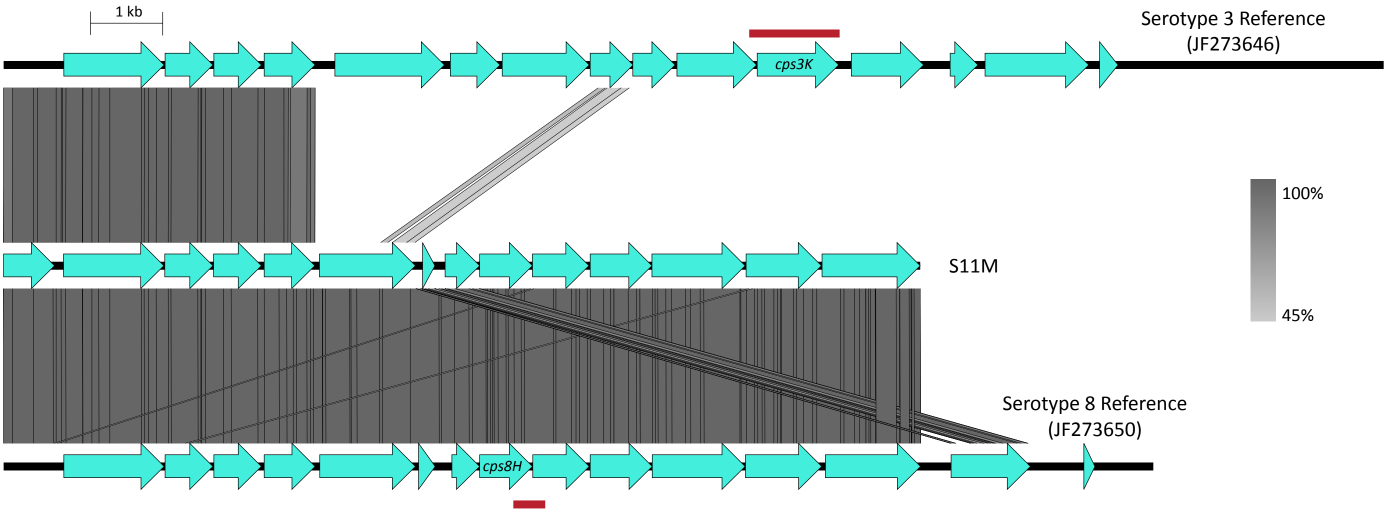

Figure S14. Comparison of *cps* loci show that strain S11M is actually serotype 8, as identified by our pipeline. Strain S11M had been typed as serotype 3 by serological methods. Short-read WGS data for strain LSW were downloaded from the Sequence Read Archive and *de novo* assembled using the A5 pipeline. The *cps* loci of a serotype 3 reference strain (top), strain S11M (middle) and a serotype 8 reference strain (bottom) were compared using the program easyFig. Serotype specific regions searched for by our pipeline are indicated in red. GenBank Accession numbers for reference sequences are provided.

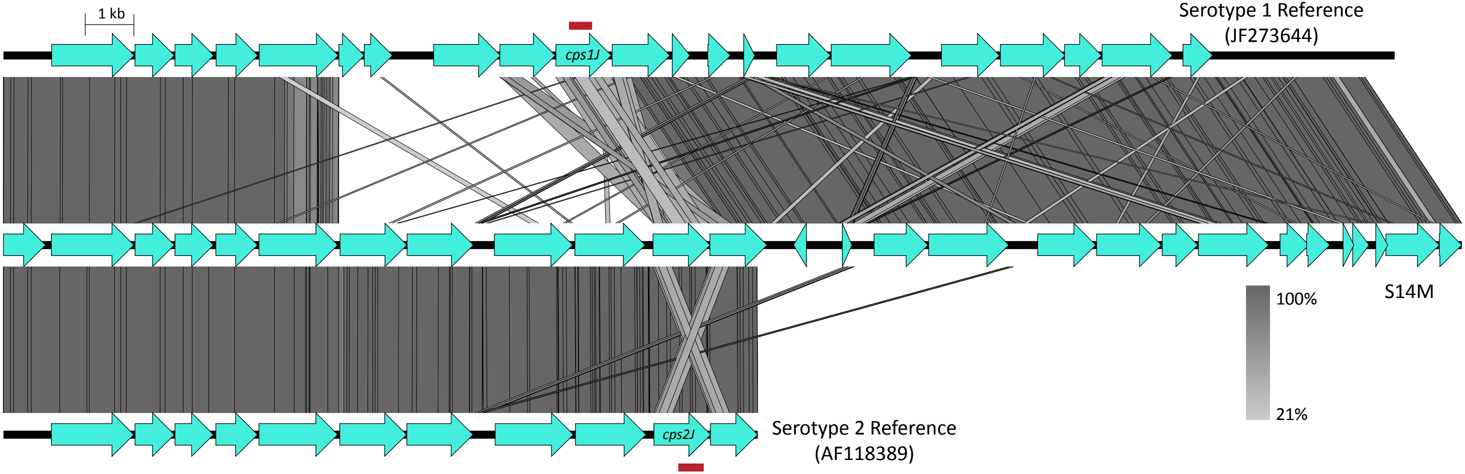

Figure S15. Comparison of *cps* loci show that strain S14M is actually serotype 15, as identified by our pipeline. Strain S14M had been typed as serotype 1 by serological methods. Short-read WGS data for strain LSW were downloaded from the Sequence Read Archive and *de novo* assembled using the A5 pipeline. The *cps* loci of a serotype 1 reference strain (top), strain S14M (middle) and a serotype 2 reference strain (bottom) were compared using the program easyFig. Serotype specific regions searched for by our pipeline are indicated in red. GenBank Accession numbers for reference sequences are provided.

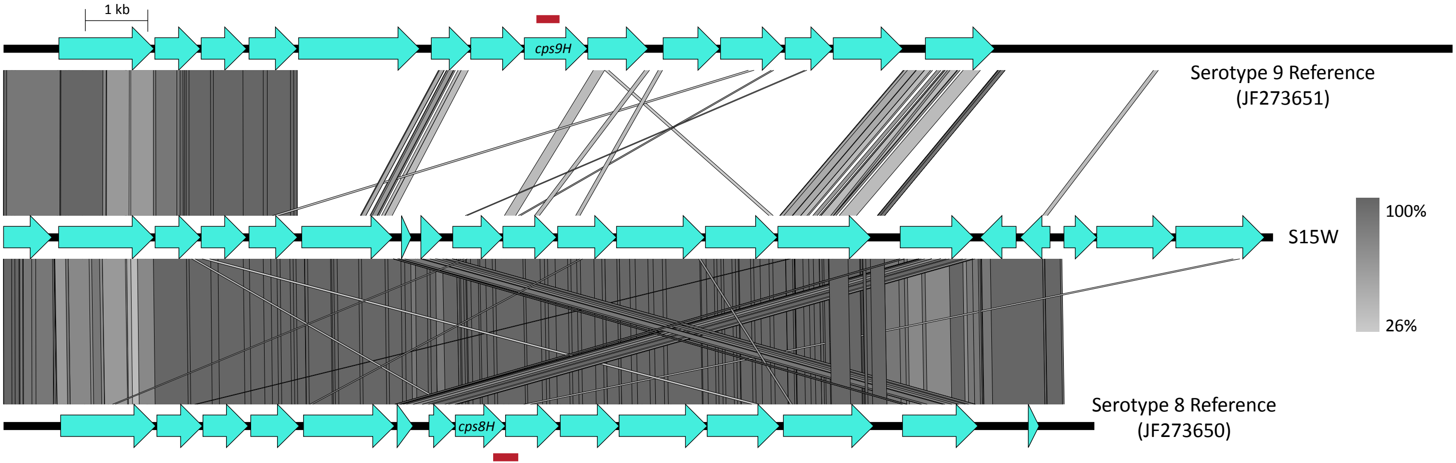

Figure S16. Comparison of *cps* loci show that strain S15W is actually serotype 8, as identified by our pipeline. Strain S15W had been typed as serotype 9 by serological methods. Short-read WGS data for strain S15W were downloaded from the Sequence Read Archive and *de novo* assembled using the A5 pipeline. The *cps* loci of a serotype 9 reference strain (top), strain S15W (middle) and a serotype 8 reference strain (bottom) were compared using the program easyFig. Serotype specific regions searched for by our pipeline are indicated in red. GenBank Accession numbers for reference sequences are provided.

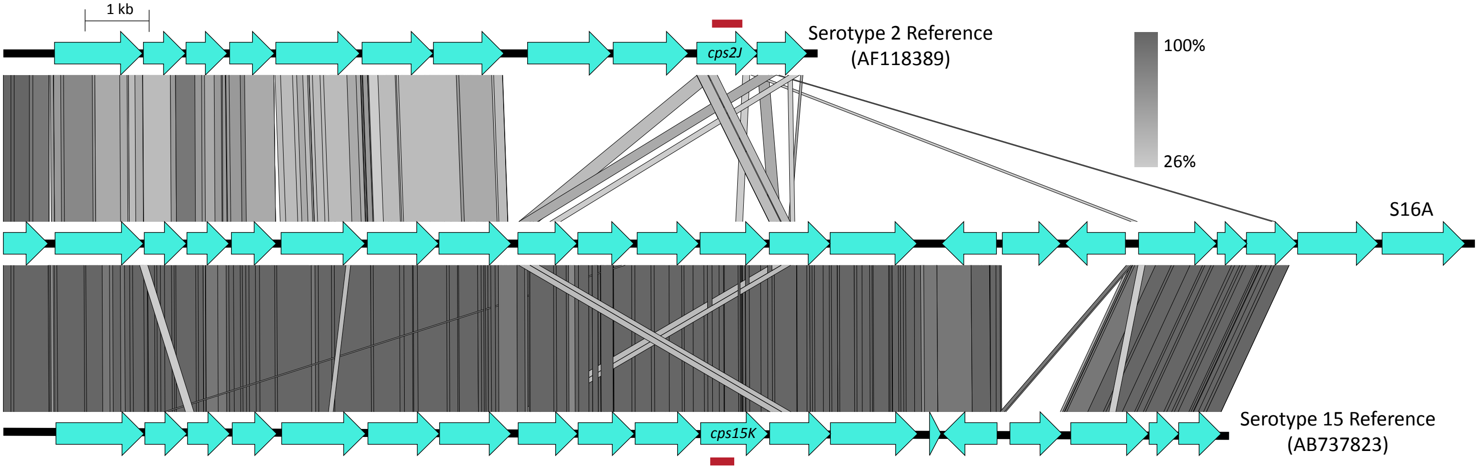

Figure S17. Comparison of *cps* loci show that strain S16A is actually serotype 15, as identified by our pipeline. Strain S16A had been typed as serotype 2 by serological methods. Short-read WGS data for strain S16A were downloaded from the Sequence Read Archive and *de novo* assembled using the A5 pipeline. The *cps* loci of a serotype 2 reference strain (top), strain S16A (middle) and a serotype 15 reference strain (bottom) were compared using the program easyFig. Serotype specific regions searched for by our pipeline are indicated in red. GenBank Accession numbers for reference sequences are provided.

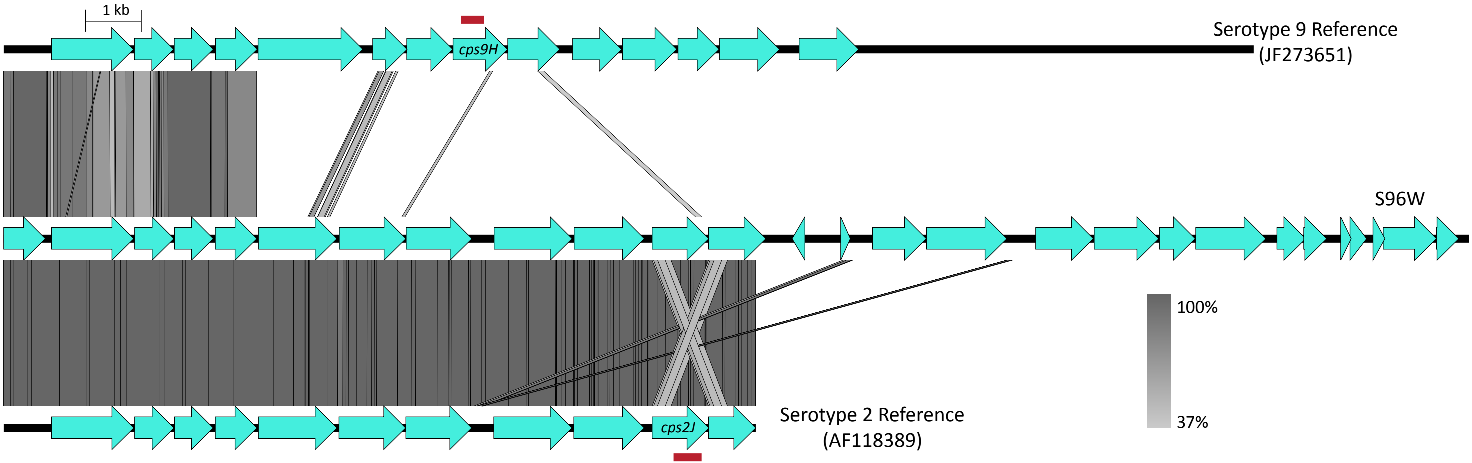

Figure S18. Comparison of *cps* loci show that strain S96W is actually serotype 2, as identified by our pipeline. Strain S96W had been typed as serotype 9 by serological methods. Short-read WGS data for strain S96W were downloaded from the Sequence Read Archive and *de novo* assembled using the A5 pipeline. The *cps* loci of a serotype 9 reference strain (top), strain S96W (middle) and a serotype 2 reference strain (bottom) were compared using the program easyFig. Serotype specific regions searched for by our pipeline are indicated in red. GenBank Accession numbers for reference sequences are provided.

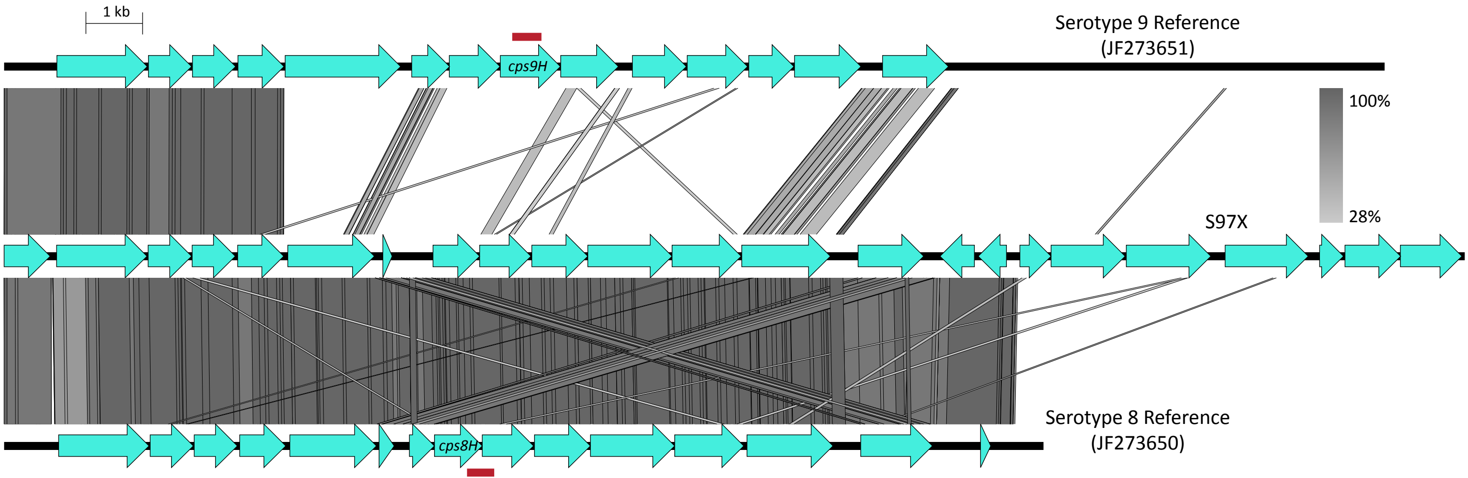

Figure S19. Comparison of *cps* loci show that strain S97X is actually serotype 8, as identified by our pipeline. Strain S97X had been typed as serotype 9 by serological methods. Short-read WGS data for strain S97X were downloaded from the Sequence Read Archive and *de novo* assembled using the A5 pipeline. The *cps* loci of a serotype 9 reference strain (top), strain S96W (middle) and a serotype 8 reference strain (bottom) were compared using the program easyFig. Serotype specific regions searched for by our pipeline are indicated in red. GenBank Accession numbers for reference sequences are provided.

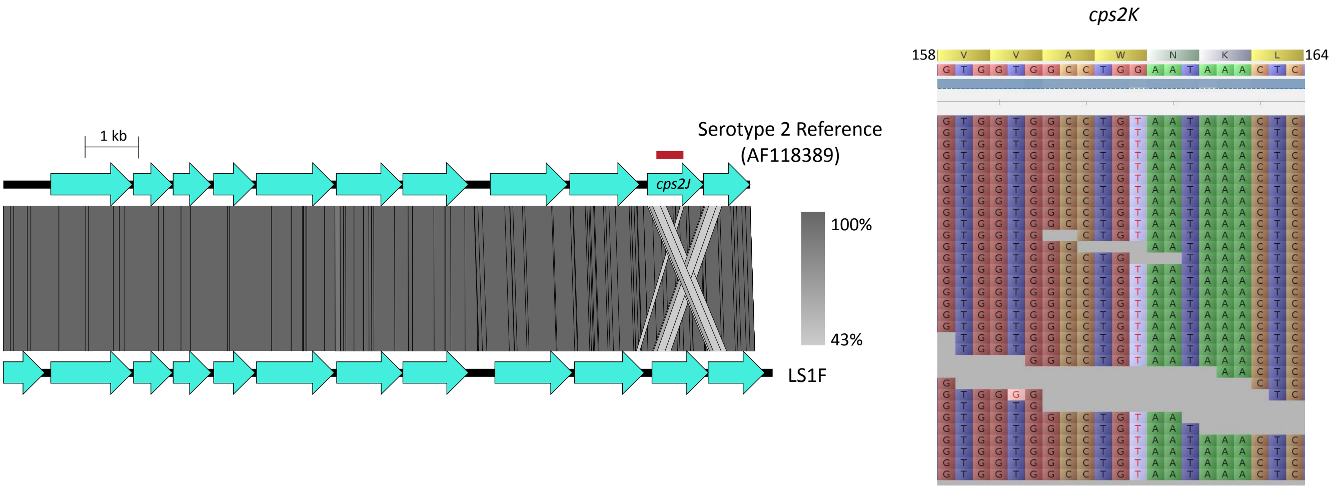

Figure S20. Our pipeline predicts that strain LS1F is actually serotype 1/2. Strain LS1F had been typed as serotype 2 by serological methods. Short-read WGS data for strain LS1F were downloaded from the Sequence Read Archive and *de novo* assembled using the A5 pipeline. The *cps* loci of a serotype 2 reference strain (top) and strain LS1F (bottom) were compared using the program easyFig, and showed a perfect match. However, short-read alignment to gene *cps2K* identified a single-nucleotide polymorphism (G483T, highlighted) in strain LS1F that we consistently associated with serotype 1/2 strains. Serotype specific regions searched for by our pipeline are indicated in red. GenBank Accession numbers for reference sequences are provided.

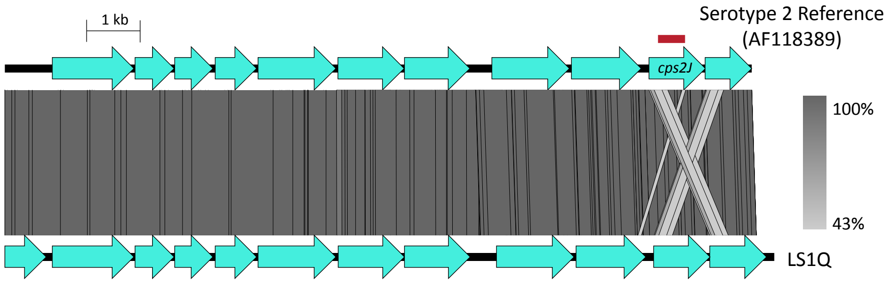

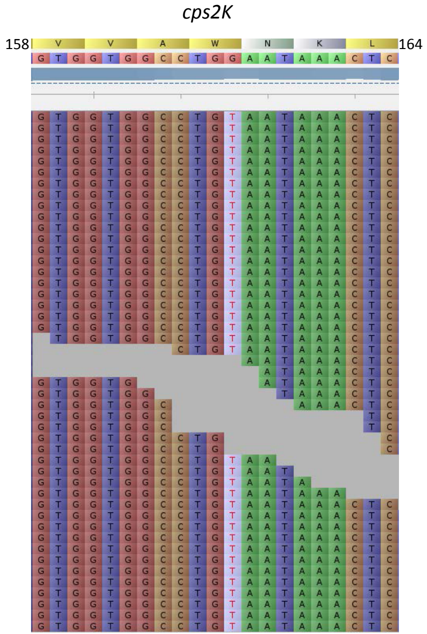

Figure S21. Our pipeline predicts that strain LS1Q is actually serotype 1/2. Strain LS1Q had been typed as serotype 2 by serological methods. Short-read WGS data for strain LS1Q were downloaded from the Sequence Read Archive and *de novo* assembled using the A5 pipeline. The *cps* loci of a serotype 2 reference strain (top) and strain LS1Q (bottom) were compared using the program easyFig, and showed a perfect match. However, short-read alignment to gene *cps2K* identified a single-nucleotide polymorphism (G483T, highlighted) in strain LS1Q that we consistently associated with serotype 1/2 strains. Serotype specific regions searched for by our pipeline are indicated in red. GenBank Accession numbers for reference sequences are provided.

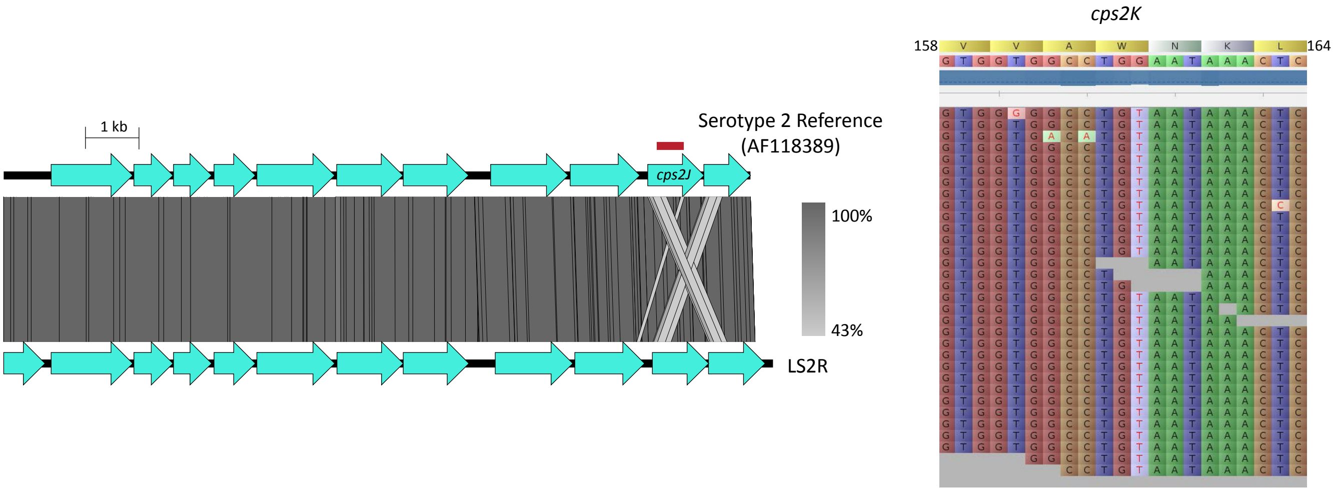

Figure S22. Our pipeline predicts that strain LS2R is actually serotype 1/2. Strain LS2R had been typed as serotype 2 by serological methods. Short-read WGS data for strain LS2R were downloaded from the Sequence Read Archive and *de novo* assembled using the A5 pipeline. The *cps* loci of a serotype 2 reference strain (top) and strain LS2R (bottom) were compared using the program easyFig, and showed a perfect match. However, short-read alignment to gene *cps2K* identified a single-nucleotide polymorphism (G483T, highlighted) in strain LS2R that we consistently associated with serotype 1/2 strains. Serotype specific regions searched for by our pipeline are indicated in red. GenBank Accession numbers for reference sequences are provided.

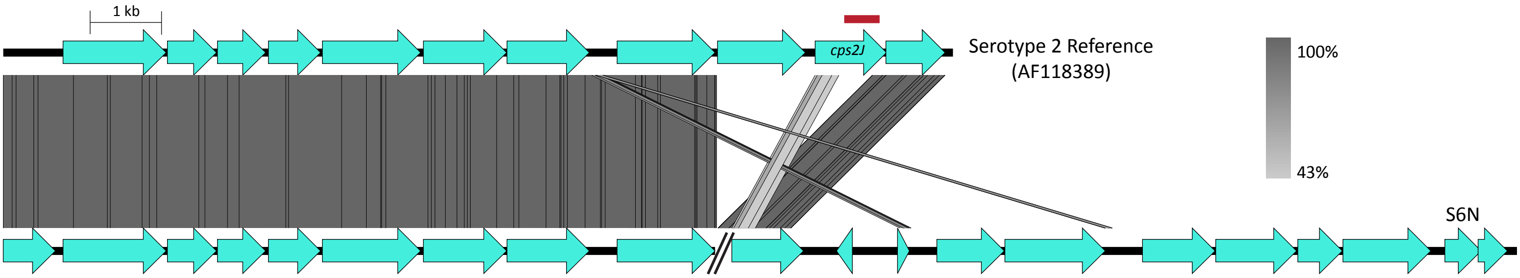

Figure S23. Comparison of *cps* loci show that strain S6N is non-typable by our pipeline Strain S6N had been typed as serotype 2 by serological methods. Short-read WGS data for strain LS6R were downloaded from the Sequence Read Archive and *de novo* assembled using the A5 pipeline. The *cps* loci of a serotype 2 reference strain (top), strain S6N (bottom) and 28 other reference strains (not shown) were compared using the program easyFig. We were unable to find sequences for genes *cpsI* and *cpsJ* in the *de novo* assemblies of strain S6N. Serotype specific regions searched for by our pipeline are indicated in red. GenBank Accession numbers for reference sequences are provided.

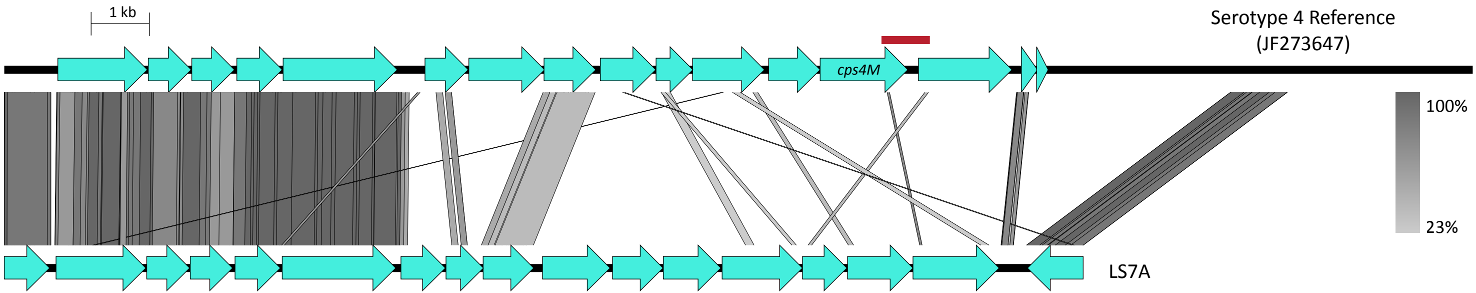

Figure S24. Comparison of *cps* loci show that strain LS7A is actually non-typable. Strain LS7A had been typed as serotype 4 by serological methods. Short-read WGS data for strain LS6R were downloaded from the Sequence Read Archive and *de novo* assembled using the A5 pipeline. The *cps* loci of a serotype 4 reference strain (top), strain LS7A (bottom) and 28 other reference strains (not shown) were compared using the program easyFig. Serotype specific regions searched for by our pipeline are indicated in red. GenBank Accession numbers for reference sequences are provided.

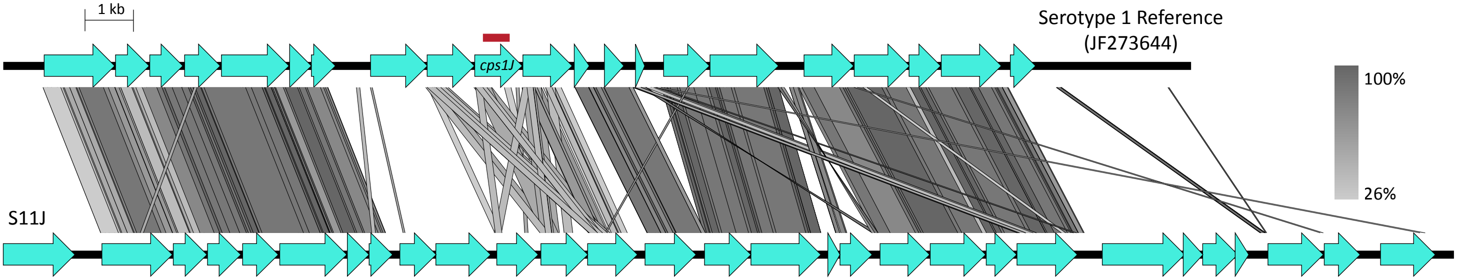

Figure S25. Comparison of *cps* loci show that strain S11J is actually non-typable. Strain S11J had been typed as serotype 1 by serological methods. Short-read WGS data for strain LS6R were downloaded from the Sequence Read Archive and *de novo* assembled using the A5 pipeline. The *cps* loci of a serotype 1 reference strain (top), strain S11J (bottom) and 28 other reference strains (not shown) were compared using the program easyFig. Serotype specific regions searched for by our pipeline are indicated in red. GenBank Accession numbers for reference sequences are provided.

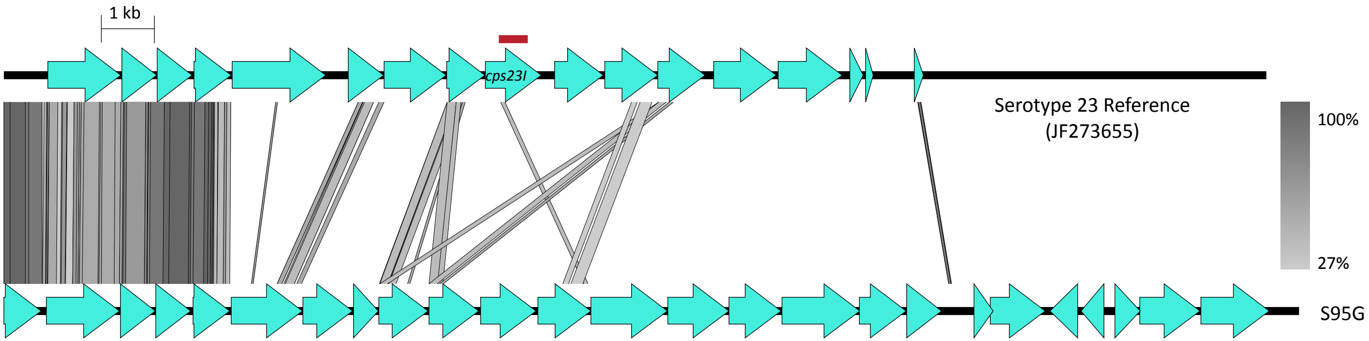

Figure S26. Comparison of *cps* loci show that strain S95G is actually non-typable. Strain S95G had been typed as serotype 23 by serological methods. Short-read WGS data for strain LS6R were downloaded from the Sequence Read Archive and *de novo* assembled using the A5 pipeline. The *cps* loci of a serotype 23 reference strain (top), strain S95G (bottom) and 28 other reference strains (not shown) were compared using the program easyFig. Serotype specific regions searched for by our pipeline are indicated in red. GenBank Accession numbers for reference sequences are provided.
